# Intra-individual polymorphism in diploid and apomictic polyploid hawkweeds (*Hieracium*, Lactuceae, Asteraceae): disentangling phylogenetic signal, reticulation, and noise

**DOI:** 10.1186/1471-2148-9-239

**Published:** 2009-09-22

**Authors:** Judith Fehrer, Karol Krak, Jindřich Chrtek

**Affiliations:** 1 Institute of Botany, Academy of Sciences of the Czech Republic, Zámek 1, 25243 Průhonice, Czech Republic; 2 Department of Botany, Faculty of Science, Charles University Prague, Benátská 2, 12801 Prague, Czech Republic

## Abstract

**Background:**

*Hieracium *s.str. is a complex species-rich group of perennial herbs composed of few sexual diploids and numerous apomictic polyploids. The existence of reticulation and the near-continuity of morphological characters across taxa seriously affect species determination, making *Hieracium *one of the best examples of a 'botanist's nightmare'. Consequently, its species relationships have not previously been addressed by molecular methods. Concentrating on the supposed major evolutionary units, we used nuclear ribosomal (*ETS*) and chloroplast (*trnT*-*trnL*) sequences in order to disentangle the phylogenetic relationships and to infer the origins of the polyploids.

**Results:**

Despite relatively low interspecific variation, the nuclear data revealed the existence of two major groups roughly corresponding to species with a Western or Eastern European origin. Extensive reticulation was mainly inferred from the character additivity of parental *ETS *variants. Surprisingly, many diploid species were of hybrid origin whilst several polyploid taxa showed no evidence of reticulation. Intra-individual *ETS *sequence polymorphism generally exceeded interspecific variation and was either independent of, or additional to, additive patterns accounted for by hybrid origin. Several *ETS *ribotypes occurred in different hybrid taxa, but never as the only variant in any species analyzed.

**Conclusion:**

The high level of intra-individual *ETS *polymorphism prevented straightforward phylogenetic analysis. Characterization of this variation as additive, shared informative, homoplasious, or unique made it possible to uncover the phylogenetic signal and to reveal the hybrid origin of 29 out of 60 accessions. Contrary to expectation, diploid sexuals and polyploid apomicts did not differ in their molecular patterns. The basic division of the genus into two major clades had not previously been intimated on morphological grounds. Both major groups are thought to have survived in different glacial refugia and to have hybridized as a result of secondary contact. Several lines of evidence suggest the data is best explained by the presence of an extinct range of variation and a larger diversity of ancestral diploids in former times rather than by unsampled variation. Extinct diversity and extensive reticulation are thought to have largely obscured the species relationships. Our study illustrates how multigene sequences can be used to disentangle the evolutionary history of agamic complexes or similarly difficult datasets.

## Background

Agamic complexes, usually consisting of few diploid and many polyploid taxa - the latter reproducing by apomixis to various degrees - are characterized by large numbers of species often exhibiting more or less continuous morphological variation. Consequently species delimitation is a challenging task, and even in the presence of expert taxonomic knowledge, does not always lead to satisfactory results. Therefore, the molecular study of species relationships in such groups is seriously hampered, and few attempts have been made to reconstruct the evolutionary history of such complexes [1].

Three or four subgenera of hawkweeds (*Hieracium *L. s.l.) have traditionally been recognized. In this study, we will focus on *Hieracium *subgen. *Hieracium *(*Hieracium *s.str.). For a review of taxonomic treatments, see [2]. *Hieracium *s.str. is a highly diverse group (500-8000 species depending on taxonomic concept) known for its notorious taxonomic complexity, which is associated with a variation in ploidy level, breeding system and supposed history of extensive hybridization [3,4]. The group consists of perennial herbs distributed mainly in temperate areas of Europe, Asia, and North America. Main centers of *Hieracium *diversity are located in the European mountains (e.g., Alps, Pyrenees, Carpathians, Balkan Peninsula) and in westernmost Asia. The species occupy forests, forest margins, various grasslands, and rocks. Polyploid (triploid and tetraploid, very rarely pentaploid) taxa [5,6] with asexual reproduction via parthenogenetic development of the unreduced egg cell (*Antennaria*-type diplospory) prevail. Polyploids are near-obligate apomicts that produce seed asexually (corresponding to the maternal genotype), but can produce pollen via meiosis to different degrees. Sexual reproduction is rare and restricted to diploid species (*2n *= 18). Diploids are mainly confined to unglaciated refugia; polyploids are also widespread in areas that had been covered by ice sheets [7]. Many taxa comprise populations of different ploidy level.

The most complete taxonomic treatment was published by Zahn [3]. He used a broad species concept and distinguished two different kinds of species: (i) 'basic' species (*species principales collectivae*, 'Hauptarten') having a unique set of morphological characters; and (ii) 'intermediate' species (*species intermediae collectivae*, 'Zwischenarten'), which combine the morphological characters of two or more 'basic' species and are generally thought to be of hybrid origin. Following this concept, we expected the diploid 'basic' species to represent major evolutionary units from which the multitude of polyploids arose. In addition, we suspected polyploid 'basic' species (i.e., taxa presumed to be of non-hybrid origin) to comprise further basal lineages, especially as the number of recent diploids is rather low (about 20 out of several hundred macrospecies). Thus, in order to cover as much as possible of the overall genetic variation present in the subgenus, we focused on all 'basic' species irrespective of their ploidy.

The 45S rDNA cistron continues to be the most popular nuclear region for species-level phylogenetic studies of plants [8] despite its acknowledged flaws [9]. The 5'-*ETS *region situated upstream of the 18S rRNA gene [10] was chosen as a nuclear marker because of its higher variability compared to *ITS *in the Asteraceae [11,12]. *ITS *showed hardly any resolution in *Hieracium *s.str. [13]. While the majority of publications based on *ETS *are focused on phylogeny reconstruction, several studies have also used this region to infer hybrid or allopolyploid origin, often in combination with *ITS *[14-21].

In sexually reproducing diploids not introgressed by other species, one would expect sequences of this multicopy region to be more or less homogeneous at the intra-individual and intraspecific level [22]. Since concerted evolution [23] tends to be slowed down in asexually reproducing organisms [24-26], apomictic allopolyploids should have retained the *ETS *variants of their respective diploid progenitors. Additivity patterns revealed by direct sequencing of nrDNA have been used to identify hybrid or allopolyploid origin in a wide range of plant groups [27-35], in one case even in a triple hybrid [36]. We therefore adopted a direct sequencing approach complemented by cloning of selected accessions.

Analysis of the *trnT-trnL *intergenic spacer of chloroplast DNA was also performed in order to establish a phylogeny of maternal lineages and to identify the maternal parent of hybrid accessions. We have recently confirmed maternal transmission of cpDNA for *Hieracium *s.str. [37].

This paper reports the first molecular phylogeny of *Hieracium *subgen. *Hieracium *based on a sampling of most of the assumed major evolutionary units. It demonstrates the unexpected hybrid origin of many of these including diploid species, provides evidence of extinct ancestral diversity and discusses the occurrence of extensive intra-individual polymorphism found in most diploid and polyploid accessions. In order to decipher the phylogenetic signal in spite of these abundant polymorphisms, we distinguished between character additivity, shared informative variation, and noise by detailed character state analysis.

## Methods

### Sampling

Zahn's [3] basic framework was used for a meaningful taxon sampling for phylogenetic analysis, as complete sampling was impractical due to the high species numbers and the unclear delimitation of 'intermediate' species. Up to three samples of each accessible 'basic', i.e., supposedly non-hybridogenous species of *Hieracium *s.str. were collected. If ploidy level varied within a species, diploid populations were included whenever available. The taxonomic concept generally follows Zahn [3] with a few exceptions: The species concept of section *Cerinthoidea *(Iberian and Pyrenean taxa) was adopted from Mateo [38], and *H. plumulosum *(*H. waldsteinii *s.l.) was treated as a separate species. Two newly described diploid species from the Balkans (*H. kittanae *and *H. petrovae*; [39,40]), diploid *H. pojoritense *- considered as an endemic species of the Eastern Carpathians by Nyárády [41], and *H. mixtum *- which was first treated as a 'basic' species by De Retz [42], followed by other authors - were also included. Altogether, we analyzed 60 accessions of 46 species. For the remaining 'basic' species *sensu *Zahn (*H. fuscocinereum *Norrlin, *H. laniferum *Cav., and *H. schmalhausenianum *Litv. & Zahn), only herbarium material more than 30 years old was available, in which the DNA was too degraded for amplification. Details of all accessions are included in the Additional file 1: Origins of individual accessions.

As outgroups for the *ETS *analyses, the most closely related genera *Pilosella*, *Hispidella*, *Andryala*, and '*Hieracium*' *intybaceum *were used [13]. For cpDNA analysis, only *Hispidella *and a *Pilosella *species with 'original' chloroplast haplotype were used as outgroups. This was because some of the *Pilosella *species, *Andryala*, and '*Hieracium*' *intybaceum *have captured chloroplasts derived from *Hieracium *due to ancient intergeneric hybridizations [13]. Vouchers of all specimens are deposited in the herbarium of the Institute of Botany in Průhonice (PRA).

### Molecular methods

DNA was isolated from fresh or CTAB-conserved leaves as described in [43]. The *ETS *region of the nuclear ribosomal DNA was PCR-amplified using the primers Ast-8 and 18 S [44]. PCR products were purified using the QIAquick PCR purification kit (Qiagen, Hilden, Germany) and sequenced (GATC Biotech, Konstanz, Germany). For each sample, both strands were directly sequenced using the PCR primers. Sequences containing more than one indel or otherwise difficult to read were cloned. Prior to cloning, the PCR products were excised from 1% agarose gels and purified with the Zymoclean Gel DNA Recovery kit (Zymoresearch, Orange, CA). The gel-purified fragments were cloned using the TOPO TA cloning kit (Invitrogen, Carlsbad, CA) following the manufacturer's instructions, but downscaled to half reactions. Approximately 24 colonies per sample were transferred into 20 μl ddH_2_0 and denaturated at 95°C for 10 min. They served as templates for subsequent PCR amplifications for sequencing. Using the Ast-8 primer, 3-15 clones per individual were sequenced (eight on average). For the *trnT*-*trnL *intergenic spacer of chloroplast DNA, PCR amplification, sequencing and indel coding were done as described previously [13]. Sequence alignments were done by eye in BioEdit [45]; they were unambiguous for both markers due to low overall variation.

### Treatment of *ETS *sequences prior to phylogenetic analysis

All *ETS *sequences contained a certain proportion of polymorphic sites. Polymorphisms were distinguished from sequencing artifacts when they occurred in both reading directions. As overall interspecific sequence divergence was low, if only single short (1-2 bp) indels were present, polymorphic sites were determined from direct sequences by reading both strands on to the indel position, and after it by confirming them in both directions via peak subtraction. Where multiple or longer indels obscured sequence reads, the samples were cloned, and the clones were used to aid peak subtraction in the electropherograms obtained by direct sequencing in order to infer polymorphic sites in regions whose readability was strongly affected by indels. Polymorphisms were represented by the IUPAC ambiguity codes.

Preliminary phylogenetic analyses of *ETS *revealed little structure apart from a monophyly of *Hieracium *[46]; all species emerged from a single basal polytomy, and the few subclades found were poorly supported. However, visual inspection of the alignment suggested the existence of two major species groups. Many accessions showed additive characters (superimposed peaks and a diagnostic indel) corresponding to positions that differed between these main groups, suggesting hybrid origin. These accessions were removed from the phylogenetic analyses. The resulting dataset was inspected for the occurrence of further additive characters that might be indicative of hybridization within each of the two species groups. Several such accessions were detected and also excluded prior to final phylogenetic analyses.

Cloned sequences - mostly from hybrids - revealed in several cases two major *ETS *variants corresponding to sequences of both major species groups. However, some were recombinant according to visual inspection, one showed a novel hybrid sequence, some showed single nucleotides occurring on the 'wrong' ribotype, and in case of strongly biased ratios of different ribotypes (or at single polymorphic positions), not all underrepresented sequences (or character states) were retrieved (Additional file 2: Patterns of *ETS *recombination). Also, the number of polymerase errors approximated the low interspecific variation in some cases. Therefore, cloned sequences were not included in the phylogenetic analyses (but see below).

In the preliminary parsimony and Bayesian analyses of the dataset from which all suspected hybrid sequences had been deleted, the effect of the remaining polymorphic sites on tree topology and branch support were assessed by including each accession twice, namely one sequence with all polymorphisms included, and a second one reflecting the major sequence type in which ambiguities were 'resolved' towards the overrepresented character state in case the latter represented at least about 70% of the total signal. In the resulting trees, both sequences of the same accession always appeared together except for *H. villosum *1029 and *H. lucidum *where they differed slightly in their placement (not shown). As these were minor effects and because of the higher information content of the dominant sequence types in both cases, for the final phylogenetic analyses, only major *ETS *sequences were used for all accessions. This also reduced computing time as well as the number of unique indels in the alignment as they were typically present in lower amounts within an individual.

In outgroup species, *Pilosella *samples produced several equally strong bands in PCR amplification that could not be eliminated by optimizations. The shortest fragment was homologous to the 5'-*ETS *of other species while the longer fragments contained duplications (variable number of large subrepeats, not shown) which had also been found in other Asteraceae [12,47]. Therefore, *Pilosella *was represented in the phylogenetic analyses by three cloned sequences of the short fragment. In *Hispidella*, intra-individual polymorphisms included one indel adjacent to two substitutions. One allele was dominating so that the second one could be inferred from direct sequencing. Both variants were included in the phylogenetic analyses.

In order to represent the complex features of the *ETS *dataset as comprehensively as possible, direct sequences of all accessions were submitted to GenBank in duplicates: (i) with all polymorphic sites included, and (ii) corrected for overrepresented character states (e.g., the major sequence types used for phylogenetic analyses). In addition, all cloned sequences were submitted; some were corrected for polymerase errors (substitutions found in only one clone and neither accounted for by direct sequencing nor present in any other taxon). Corrected clones are indicated, e.g., as 'clone 1c' in the sequence description line of the submitted sequences; recombinant sequences are indicated as such in the notes. [GenBank:EU821363-EU821419, EU867566-EU867709, and FJ858089-FJ858133 (*ETS*), and GenBank:EU867710-EU867763 (*trnT-trnL*)]. Eight additional *trnT-trnL *sequences (for two outgroup and six *Hieracium *accessions) were adopted from [13].

### Phylogenetic analyses

Maximum parsimony (MP), maximum likelihood (ML) (PAUP* V4.0b10, [48]), and Bayesian inference (MrBayes V3.1.2, [49,50]) were applied for phylogenetic analyses of the *ETS *and *trnT*-*trnL *datasets.

For MP analysis of the *ETS *region, single-base gaps were treated as a 5^th ^character state, longer indels as a single character, and remaining ambiguous bases as polymorphisms. Heuristic searches were performed with 1,000 random sequence addition replicates, saving no more than 100 trees of length greater than or equal to 1 per replicate and TBR branch swapping. Bootstrapping was done with the same settings, but without branch swapping. Prior to ML analysis, the model of molecular evolution best fitting to the data was determined with Modeltest version 3.5 [51]. A HKY+G model was found in hierarchical Likelihood Ratio Tests (hLRTs) which was used for ML analyses with the estimated parameter settings. Heuristic searches were done with one random addition sequence replicate and TBR branch swapping; 1,000 bootstrap replicates were performed without branch swapping. For Bayesian analyses, the same basic model parameters determined by Modeltest (two substitution rates and gamma distribution) were used. Two replicate analyses with four chains each were performed with the default parameters and computed for 3 million generations, sampling every 1,000^th ^tree. All statistical parameters indicated that convergence was reached. The first 1,000 trees per run were discarded as burn-in, and the remaining 4,002 trees were summarized.

For analyses of cpDNA, MP analysis was performed as described above, treating indels as single events. As sequence divergence was low, character state changes were mapped onto the branches by hand, and homoplasies were identified based on the alignment. For ML analysis, the F81 model determined in hLRTs was applied. Accordingly, Bayesian analyses were run using one substitution rate and equal rates as priors, all else as indicated above.

### Evaluation of intra-individual polymorphism

Intra-individual polymorphism in multicopy sequences results from the presence of more than one ribotype within a particular genome accompanied by incomplete homogenization of the different variants by concerted evolution. If meaningful patterns can be distinguished from stochastic ones, intra-individual polymorphisms can add considerably to the understanding of evolutionary processes. As potentially meaningful polymorphisms we considered (i) sites that consisted of different character states (nucleotides or indels) that were monomorphic in other sequences of the dataset (character additivity), and (ii) sites showing the same alternative character states in more than one accession, but one character state was missing in the rest of the dataset (shared polymorphisms). The latter can either be meaningful (in the case of shared ancestral polymorphism) or not (if they are homoplasious). We distinguished putative homoplasious polymorphisms according to (i) geography - accessions for which recent or past overlap of distribution areas is highly unlikely; (ii) phylogeny - polymorphism-sharing accessions belonging to divergent clades, or hybrids of certainly different origin; and (iii) singularity - no further evidence for grouping these particular accessions (or accession groups) could be found. In most cases, more than one of these criteria applied. Unique (accession-specific) polymorphic sites represented a further kind of uninformative variation. A comprehensive list of sites along with the accessions in which the polymorphisms occurred and their interpretation according to the criteria described above is given in Additional file 3: Summary of intra-individual polymorphisms. To facilitate reproducibility of these inferences, an alignment with the corresponding positions is also provided (Additional file 4: Alignment of *Hieracium ETS *sequences).

### Identification of further lineages

Detailed analysis of shared polymorphisms revealed several groups of accessions that were characterized by different sets of consistently shared polymorphisms. In order to analyze the origin of this variation in more detail, all samples for which meaningful patterns emerged after analysis of intra-individual polymorphism were also cloned and sequenced as above. Clones were inspected visually, and all non-recombinant clones containing further signal (i.e., exceeding accession-specific variation, see Additional file 2: Patterns of *ETS *recombination) were subjected to additional phylogenetic analyses under the same conditions as described above.

## Results

### Intra-individual polymorphism in the *ETS *region

A surprisingly high level of intra-individual polymorphism in *ETS *sequences was found in many accessions irrespective of ploidy, ranging from one to 37 per sample (12 on average). In the whole ingroup (Additional file 4: Alignment of *Hieracium ETS *sequences), intra-individual polymorphisms concerned 196 aligned positions, exceeding the number of variable sites based on substitutions (120). At 30 of these 196 positions, two different kinds of polymorphism were found (e.g., Y and K); at five further positions, even three different ones were observed (e.g., Y, K and S). Thus, the total number of different intra-individual polymorphisms was 236 out of 573 aligned characters. A complete list of polymorphic positions, their occurrence in particular accessions, and their classification as unique, shared (homoplasious or informative), or additive is given in Additional file 3: Summary of intra-individual polymorphisms.

**Figure 1:**
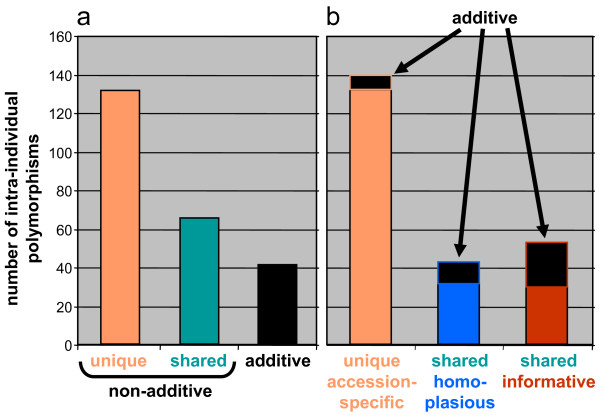
**Categories of intra-individual polymorphism**. The large amount of uninformative (unique) and potentially misleading (homoplasious) versus meaningful (additive, shared informative) variation is illustrated. a) Discrimination of non-additive (one of the character states is missing in the whole dataset, e.g., T and Y are present, but no C) from additive polymorphisms (e.g., T, Y as well as C are present). b) Further discrimination of polymorphisms shared by two or more accessions into homoplasious or informative variation, and distribution of polymorphisms that show character additivity for all categories.

At most of the polymorphic sites (unique and shared), one of the alternative character states was lacking across the whole dataset while true character additivity was comparatively rare (Figure 1a). A large proportion of the polymorphisms was accession- or species-specific. Most of this variation was uninformative, but a few unique polymorphisms showed character additivity (Figure 1b) and indicated hybrids of unique origin (see below). Among the polymorphisms shared between accessions of different species, some were shared among apparently unrelated species (according to the phylogeny), or between reasonable groups of accessions and single or multiple outliers. These polymorphisms were considered as homoplasious according to the criteria specified in the Methods. 'Reasonable groups of accessions' refers to assemblages that were supported by more than one shared polymorphism and usually also by other evidence (e.g., synapomorphic substitutions, phylogenetic position, geography). In several of these cases, character additivity applied to one of the 'reasonable' subgroups (Figure 1b) which often comprised many accessions. Thus, meaningful information could in some cases also be retrieved from homoplasious positions by a detailed evaluation of the patterns. Shared polymorphisms for which no obvious evidence for homoplasy was found were considered as potentially informative. A comparably large part of them was additive (Figure 1b). Typically, the same or similar sets of accessions shared further polymorphisms and were usually also supported by other evidence (e.g., phylogenetic position, geography). Thus, informative intra-individual polymorphism could be retrieved from an excess of uninformative or even misleading variation by detailed character state analyses. Additive and shared informative variation was crucial for inferring reticulation patterns or added to the phylogenetic information content of the data (see below).

### *ETS *phylogeny

Interspecific variation of the *ETS *region in *Hieracium *was rather low; maximal sequence divergence based on substitutions was 4.6% *p*-distance (compared to 1.9% in *ITS*, not shown). Seven short indels (1-10 bp) occurred, six of them represented intra-individual polymorphisms, only one differed between accessions.

**Figure 2:**
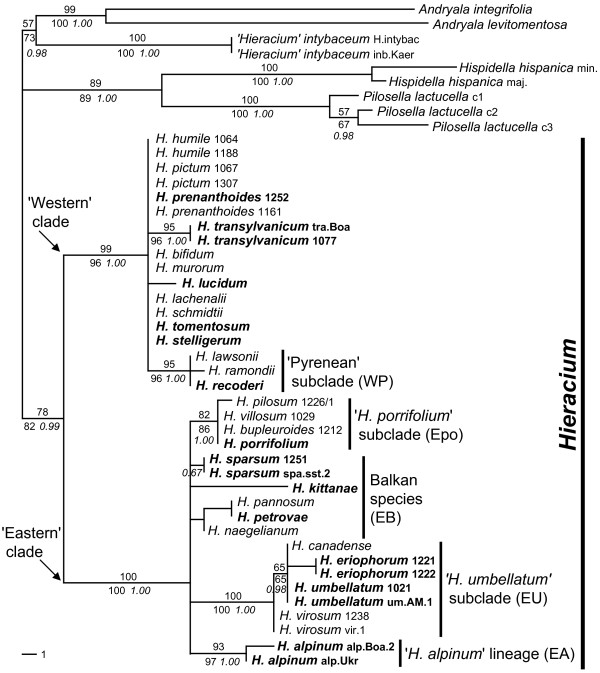
**Molecular phylogeny of major evolutionary units of *Hieracium *based on *ETS *sequences**. One out of 256 equally parsimonious trees is shown (417 steps, ri = 0.947, ci = 0.940; 153 variable characters of which 89 were parsimony informative) with bootstrap support indicated above the branches. The strict consensus tree topology corresponds to branches with support values in the MP analysis. Bootstrap values for ML and posterior probabilities for Bayesian analyses are given below the branches. Diploid *Hieracium *species are indicated in boldface. Four accessions (*H. prenanthoides *1252 and 1161, *H. lachenalii*, *H. lucidum*) are included in these analyses in spite of their inferred hybrid origin, because *ETS *sequences of the first three were 'Western' apart from 2-3 polymorphic sites, and the dominant sequence of *H. lucidum *was also 'Western'.

Phylogenetic analyses of the dataset from which all inferred hybrid accessions had been excluded based on character additivity resulted in basically the same tree under different optimality criteria (Figure 2). A basal split into two well-supported major clades was found, one composed mostly of species with Western, the other one of taxa with mainly Eastern European distribution. Widespread or Central European taxa fell into one or other cluster. Both major clades were characterized by basal polytomies from which a few well-supported subclades emerged.

All species of the 'Eastern' group that occurred at unresolved positions are from Southeastern Europe, mostly from the Balkans (*H. naegelianum*, *H. pannosum*, *H. petrovae*, *H. sparsum*, *H. kittanae*) (Figure 2). One lineage emerging from the basal polytomy of the 'Eastern' clade consisted of two diploid accessions of *H. alpinum*. While *H. alpinum *s.l. is a widespread species, diploid populations occur only in the Eastern and Southern Carpathians [52] consistent with an eastern origin of that species. The '*H. porrifolium*' subclade consisted of the alpine species *H. pilosum*, *H. villosum*, *H. bupleuroides*, and *H. porrifolium*. The first three are Central European polyploid taxa; *H. porrifolium *is the only diploid species in this subclade. This taxon is restricted to the Southeastern Alps - a known glacial refuge area. Species of the '*H. porrifolium*' group occur only in rock crevices on limestone and have a very similar ecology. The best supported group ('*H. umbellatum*' subclade) consisted of the tall-growing perennials *H. virosum*, *H. umbellatum*, *H. eriophorum*, and *H. canadense*. Their distribution extends from Siberia to North America. The kind of involucrum in these species, an important character complex for *Hieracium *taxonomy, resembles that of other species with exclusively Eastern European distribution. *Hieracium umbellatum *is the most widely distributed diploid.

In the 'Western' cluster, subclades emerging from the basal polytomy were a branch composed of three Pyrenean taxa ('Pyrenean' subclade) and a lineage consisting of two accessions of *H. transylvanicum*. All species with exclusively or mainly Pyrenean or Western Alpine distribution belonged to the 'Western' clade, some of them may be remnants of previously much larger populations. In addition, diploid populations of *H. prenanthoides *are also restricted to the Southwestern Alps, indicating western origin. One species, *H. lucidum*, is endemic to Northwestern Sicily and is only known from a single relict population. Widespread or Central European species whose accessions fell into the 'Western' clade were *H. schmidtii*, *H. murorum*, and *H. bifidum*. The accession of the widespread species *H. lachenalii *used, was apparently introgressed by a species from the ('Eastern') '*H. umbellatum*' subclade as indicated by its chloroplast DNA (see below). The only species not fitting into the 'Western' clade according to its distribution was *H. transylvanicum *which occurs in the Carpathians, the Eastern Alps and the Northern Balkans. Its large genome size (about 10% higher than that of 'Western' species) may also suggest an eastern origin (for details, see [46]). We therefore cannot exclude that this is a similar case to *H. lachenalii*, however, the chloroplast haplotype of *H. transylvanicum *was unique and did not allow an assignment to any ('Eastern'?) taxon.

Diploid accessions - all were sexual and self-incompatible - were spread all over the tree and occupied basal as well as derived positions within the major clades (Figure 2). The same was true for polyploid (triploid or tetraploid) accessions, which were experimentally confirmed as apomictic. Accessions included in the 'Western' cluster had significantly lower DNA content (1Cx values corrected for ploidy) than those of the 'Eastern' clade (3.66 ± 0.21 pg versus 4.03 ± 0.19 pg, see Additional file 5: Species/accessions, their origin, cytotype, *ETS *and cpDNA features). Details of reproduction, ploidy, and genome size are given in a parallel paper [46]. Thus, the major clades revealed by phylogenetic analysis of the *ETS *region coincide with geographic patterns and with significant genome size differences (~10% on average).

### Chloroplast haplotypes

**Figure 3:**
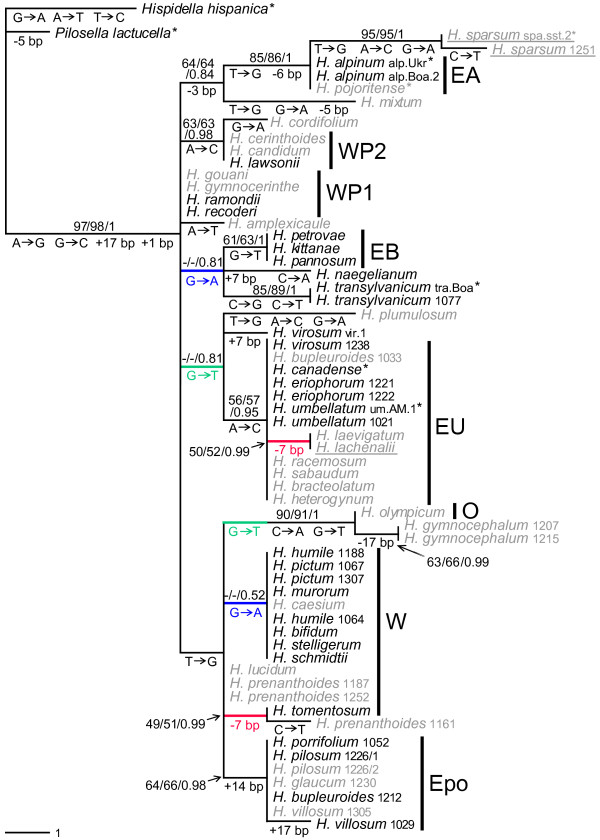
**Phylogenetic analysis of the chloroplast *trnT-trnL *intergenic spacer**. One out of 60 equally parsimonious trees is shown (44 steps, ri = 0.972, ci = 0.988; 37 variable characters of which 21 were parsimony informative) whose topology exactly matches that of ML and Bayesian analyses including unsupported groups. Character state changes are shown below the respective branches, identical colors indicate homoplasious mutations. Bootstrap values for MP/ML and Bayesian posterior probabilities are above branches. Hybrid accessions are in grey, those for which hybrid origin was only implied by cpDNA are underlined. Asterisks after species names: sequences adopted from [13]. Acronyms of particular haplotype groups refer to Additional file 5: Species/accessions, their origin, cytotype, *ETS *and cpDNA features; a subset corresponds to the subclades and species groups in Figure 2.

Variation of the *trnT*-*trnL *intergenic spacer of chloroplast DNA was expectedly lower (max. 2.12% *p*-distance) than that of the nuclear *ETS*. Figure 3 summarizes the results of the phylogenetic analyses for this marker. The tree also allows to determine the number of substitutions and indels that distinguish between particular sequences. Three homoplasious mutations occurred. Two of these were substitutions in rather basal positions (G → T, G → A) that accounted for major rearrangements in equally parsimonious trees. These branches were not supported in any of the analyses. In contrast, a 7 bp-deletion was always derived from haplotypes of either *H. lucidum *or *H. umbellatum *(or sequences identical to these), with a minimum of three substitutional steps (as in the tree shown), or with up to five steps separating *H. lachenalii*/*H. laevigatum *from *H. tomentosum*/*H. prenanthoides *1161. Both lineages found significant support in the Bayesian analysis. As this mutation involved a tandem repeat, we assume parallel deletion events, especially as part of the same motif was independently lost in *H. mixtum*. For the assignment of haplotype groups, homoplasious substitutions were ignored.

Bootstrap support was generally low - if haplotypes differ only by a single mutation, it cannot be higher on principle [53]. We therefore concentrated only on particular haplotypes (often identical sequences) and on conservative inferences we considered as unequivocal. No species relationships were inferred from this marker. Despite the low bootstrap support, all but the three above-mentioned mutations were free of homoplasy (which is reflected by very high retention and consistency indices in the parsimony analysis, see Figure 3) and can therefore be considered as diagnostic. Posterior probabilities in Bayesian analysis were usually significant (≥ 0.95).

The inferred haplotype groups (Figure 3) generally matched the particular clades or species groups found in the *ETS *analyses (Figure 2). Taxa of the 'Pyrenean' lineage were subdivided into two haplotype groups separated by a single substitution; *H. naegelianum *had a unique haplotype and was excluded from the 'Balkan' group. Shared chloroplast haplotypes (usually identical sequences) allowed inference of the maternal origin of most hybrid accessions and always matched the subclades of one parent previously inferred from the *ETS *dataset (see also below). In addition, a few rather derived unique chloroplast haplotypes occurred, especially in hybrid accessions whose exact parentages could not be inferred from the *ETS *data. Only in two cases did cpDNA clearly contradict the phylogenetic position of the species revealed by *ETS*. One was *H. lachenalii*, which showed a 'Western' *ETS*, but its cpDNA corresponded to species of the '*H. umbellatum*' clade, a derived 'Eastern' lineage. The taxon also shows some morphological features of species belonging to the '*H. umbellatum*' group. However, no trace of such an introgression was found in the *ETS*. The genome size of *H. lachenalii *was about 5% higher than the usual values of other 'Western' clade species (Additional file 5: Species/accessions, their origin, cytotype, *ETS *and cpDNA features). Therefore, chloroplast capture indicative of cryptic hybridization between a diploid maternal parent of the '*H. umbellatum*' clade and a 'Western' pollen donor with almost complete homogenization of *ETS *towards the 'Western' parent (see also Table 1) seems to be the most likely explanation for the discrepancy. The other case was *H. sparsum*. The cpDNA haplotypes of both analyzed accessions were almost identical and unique, they differed by many mutations from all other Balkan species. Furthermore, they were apparently derived from an '*H. alpinum*' haplotype. This result was less easy to interpret. It is possible that either a chloroplast capture event occurred very early in the history of the species or that *H. sparsum *is derived from an unknown maternal species with '*H. alpinum*' cpDNA (for more details, see also Additional file 1: Origins of individual accessions).

**Table 1:** Positions distinguishing 'Eastern' and 'Western' clades and signatures of selected hybrid accessions

**Clades and particular accessions**	**Diagnostic sites differing between major clades (position in alignment)**														
	**187**	**194**	**197**	**233**	**244**	**252**	**262**	**281**	**307**	**368**	**446**	**461**	**478**	**486**	**488**
**'Western' clade**	**G**	**C**	**T**	**C**	**G**	**C**	**T**	**T**	**G**	**C**	**G**	**T**	**T**	**A**	**T**
*'Eastern' clade*	*T*	*A*	*C*	*T*	*T*	*T*	-	*C*	*A*	*T*	*A*	*A*	*C*	*T*	*A*
Interclade hybrids^1^	K	M	Y	Y	K	Y	-/T	Y	R	Y	R	W	Y	W	W
*H. prenanthoides *1252	**G**	**C**	**T**	**C**	**G**	**C**	**T**	**T**	**G**	**C**	**G**	**T**	Y	W	W
*H. prenanthoides *1161	**G**	**C**	**T**	**C**	**G**	y	**T**	**T**	**G**	**C**	**G**	**T**	Y	W	W
*H. prenanthoides *1187	k	m	y	y	k	y	-/t	y	r	y	r	w	Y	W	W
*H. prenanthoides *1187 dominant peaks	**G**	**C**	**T**	**C**	**G**	**C**	**T**	**T**	**G**	**C**	**G**	**T**	Y	W	W
*H. lachenalii*	**G**	**C**	**T**	**C**	**G**	y	**T**	**T**	r	**C**	**G**	**T**	**T**	**A**	**T**
*H. mixtum* dominant peaks	**G**	**C**	*C*	**C**	**G**	**C**	-	**T**	*A*	*T*	**G**	**T**	*C*	*T*	*A*
*H. plumulosum* dominant peaks	*T*	*A*	*C*	*T*	*T*	*T*	-	*C*	*A*	*T*	*A*	*A*	*C*	*T*	*A*

^1 ^Interclade hybrids that show character additivity at all 15 sites comprise *H. amplexicaule*, *H. bracteolatum*, *H. caesium*, *H. glaucum*, *H. gouani*, *H. gymnocephalum *(both accessions), *H. heterogynum*, *H. laevigatum*, *H. mixtum*, *H. olympicum*, *H. pilosum *1226/2, *H. plumulosum*, *H. prenanthoides *1187, *H. racemosum*, *H. sabaudum*, and *H. villosum *1305.

Lower case letters indicate unequal representation of superimposed peaks.

'Western' character states are in boldface; 'Eastern' in italics.

A compilation of accessions, their geographic origin, ploidy/DNA content, *ETS *features, and cpDNA variant (as depicted in Figures 2 and 3) is given in Additional file 5: Species/accessions, their origin, cytotype, *ETS *and cpDNA features.

### Reticulation within 'Eastern' and 'Western' clades

Two accessions showing within-'Eastern' clade hybrid origin were identified by additivity patterns. One was the diploid *H. pojoritense*, which was comprised of an '*H. umbellatum*' group sequence (resulting in seven additive characters at positions 232, 326, 349, 425, 426, 459, and 479) and the '*H. alpinum*' ribotype (responsible for another four additive sites at positions 120, 123, 180, and 340; Additional file 4: Alignment of *Hieracium ETS *sequences); the latter *ETS *variant was strongly predominating in the hybrid. Its chloroplast DNA also corresponded to *H. alpinum *(Figure 3). The second was an accession of triploid *H. bupleuroides *(1033) that turned out to be 'pure' *H. bupleuroides *introgressed by the '*H. umbellatum*' group: Character additivity for the seven diagnostic sites of the latter plus two sites reflecting the synapomorphic substitutions of the '*H. porrifolium*' clade (positions 254 and 367) indicated members of these two subclades as parents. In addition, at two of the '*H. umbellatum*' clade diagnostic sites, a 'pure' accession of *H. bupleuroides *(1212) showed unique polymorphisms whose alternative character states differed from those of the '*H. umbellatum*' group. The particular positions (425, 426) in *H. bupleuroides *1033 showed the corresponding triple peaks. This plant resembled *H. umbellatum *in some morphological characters, and its chloroplast DNA also corresponded to the '*H. umbellatum*' haplotype. Another taxon with putative hybrid origin within the 'Eastern' group was *H. sparsum *(both analyzed accessions). In this case, the only evidence for potential reticulation is based on the discrepancy between nuclear and chloroplast DNA (see above).

Four within-'Western' clade accessions of putative hybrid origin were identified; all had similar origins. Species of the 'Pyrenean' subclade differed by 2-3 synapomorphic substitutions (at positions 171, 259, and 485) from the majority of 'Western' clade species that occurred at unresolved positions. Four Pyrenean species showed the corresponding character additivity while lacking patterns that would link them to any other taxa: *H. cerinthoides*, *H. cordifolium*, *H. candidum*, and *H. gymnocerinthe*. Their cpDNA haplotypes were either identical to those of taxa of the 'Pyrenean' subclade or were slightly derived from these (Figure 3).

### Hybrids between major clades: a multitude of origins

Most hybrid accessions proved to have originated from different combinations of 'Eastern' *and *'Western' clade species. The major clades differed by 14 substitutions and one diagnostic 1 bp-indel. These sites were additive in the interclade hybrids (Table 1). In most cases, either equal or biased amounts of the respective character states of one or the other major group were found throughout the entire sequence. Thus, relative peak height and/or relative number of clones usually corresponded to parental *ETS *variants present in either equal or different amount in the hybrid accessions.

Apart from the general contribution of 'Eastern' and 'Western' lineages, one or both of the respective parental taxa of the interclade hybrid accessions could often be narrowed down to particular subclades, sometimes even to the genotype of a particular parental accession: In addition to the 15 polymorphisms representing the differences between the major clades, contribution from the '*H. umbellatum*' subclade to interclade hybrids could be inferred from seven additional diagnostic characters (see above). This was the case for *H. racemosum*, *H. sabaudum*, *H. laevigatum*, *H. bracteolatum*, and *H. caesium*. All of these except *H. caesium *('Western' cpDNA haplotype) also had a chloroplast haplotype corresponding to that of the '*H. umbellatum*' clade. In *H. heterogynum*, only two of the seven additive characters were present in *ETS*. The cpDNA haplotype of that accession also corresponded to the '*H. umbellatum*' haplotype. Almost complete 'Western' *ETS*, but '*H. umbellatum*' cpDNA was found in *H. lachenalii *(see also above). Another hybrid taxon, *H. glaucum*, was composed of an unidentified 'Western' clade parent and a member of the ('Eastern') '*H. porrifolium*' group which also donated the cpDNA. The same pattern was found in one accession of each *H. pilosum *and *H. villosum *(1226/2 and 1305), whose 'pure' accessions (1226/1 and 1029) belonged to the '*H. porrifolium*' clade. An additive polymorphism (position 21) occurring in the hybrid accession *H. pilosum *1226/2 reflected a unique substitution of *H. pilosum *1226/1 which was collected from the same site. The genome constitution of the analyzed accessions of *H. amplexicaule *and *H. gouani *was a combination of an unidentified 'Eastern' clade taxon with a member of the 'Pyrenean' subclade. The cpDNA of *H. gouani *corresponded to one of the Pyrenean haplotypes, the *H. amplexicaule *sequence was probably derived from that variant (Figure 3). For four accessions of three hybridogenous taxa with contribution of both clades (*H. gymnocephalum*, *H. olympicum*, and *H. plumulosum*), no particular species subgroup could be identified for either parent based on character additivity. They also had unique chloroplast haplotypes.

Two particularly interesting cases with respect to *ETS *patterns were the interclade hybrids *H. prenanthoides *and *H. mixtum*. For *H. prenanthoides*, three accessions were analyzed. One of the triploids (1187) was a hybrid between the major clades with the 'Western' ribotype strongly predominating (Table 1) and four out of seven additional polymorphisms reflecting the '*H. umbellatum*' clade (see also cloned sequences for this accession, Additional file 2: Patterns of *ETS *recombination). The other two accessions seemed at first to have an ordinary 'Western' sequence. However, while the diploid 1252 and the triploid 1161 did not show additivity at 12 out of 15 positions distinguishing between the clades, the last three positions at the 3'-end of the *ETS *were strongly additive (equal representation of both character states). In the triploid 'obvious' hybrid 1187, these same three sites showed equal amounts of superimposed peaks at these positions compared to the strongly biased proportion of 'Eastern' and 'Western' ribotypes in the rest of the sequence (Table 1). Thus, it seems that the diploid represents an interclade hybrid that had lost most of the 'Eastern' ribotype, probably by intragenomic recombination. The same signature of strong character additivity at the 3'-end plus several *H. prenanthoides*-specific polymorphic sites occurred in both triploid accessions indicating that the diploid *H. prenanthoides *gave rise to both triploids. Accession 1187 appears to have originated by subsequent hybridization with the '*H. umbellatum*' lineage. Another interclade hybrid, *H. mixtum*, was unique in showing a mixture of strongly predominating 'Eastern' *or *'Western' character states (see also Additional file 2: Patterns of *ETS *recombination). Strongly overrepresented character states alternated seven times between the diagnostic 'Eastern' and 'Western' character states (Table 1) which indicates a novel hybrid sequence most probably generated by gene conversion.

### Inferences from shared informative polymorphisms

Apart from intra-individual polymorphism reflecting *ETS *character state additivity and thus shared by several accessions because of similar hybrid origin, some shared informative variation (Figure 1b) also existed at positions where one of the alternative character states was missing in the dataset. In this section, we will focus on examples where several such polymorphisms (classified as shared informative) were shared by different taxa in order to retrieve meaningful information exceeding the results inferred from character additivity and phylogenetic analyses.

Species of the '*H. porrifolium*' clade were, in addition to synapomorphic substitutions, also linked by shared polymorphisms. Some of the latter indicate a closer relationship between *H. porrifolium *and *H. bupleuroides *(positions 194 and 375) as well as between *H. villosum *and *H. pilosum *(positions 351 and 369) which corresponds to the morphology and sectional classification of these species.

Shared polymorphic sites between a particular accession of *H. umbellatum *(um.AM.1) and *H. eriophorum *(positions 393 and 412) could indicate that the latter species (endemic to Southwestern France) is a young derivative from a similar genotype or from a common ancestral one with *H. umbellatum*. This would also explain the eastern origin inferred from phylogenetic analysis despite the Western European distribution of *H. eriophorum*. The same two polymorphisms were seen in *H. pojoritense *whose *ETS *sequence was additive for the '*H. umbellatum*' clade and the *H. alpinum *lineage. They narrowed down the paternal parent of *H. pojoritense *to either *H. eriophorum *(which can be excluded on its geographic distribution and endemic status) or to a genotype similar to *H. umbellatum *um.AM.1.

Apart from synapomorphic substitutions, the 'Pyrenean' subclade also differed from most 'Western' species by 2-3 shared polymorphisms (positions 171, 317, and 376). The same also occurred in four Pyrenean taxa that occupied an intermediate position between basal 'Western' species and the 'Pyrenean' subclade and could represent ancestral variation. According to character additivity, they may have hybrid origin (see above). However, an alternative explanation could be that character states identifying the 'Pyrenean' lineage originated as polymorphisms retained in the four putative hybrids. The taxa with more homogeneous *ETS *could then be younger derivatives in which the alternative nucleotides became fixed by concerted evolution. In any case, shared polymorphisms along with geographic vicinity, rather restricted distribution ranges and 'Pyrenean' chloroplast haplotypes indicate a particularly close relationship of these seven taxa.

**Figure 4:**
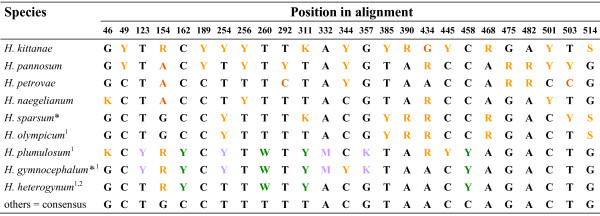
**Shared polymorphisms in species from the Balkans**. * Two accessions of this species are identical for these characters (except for *H. gymnocephalum *1215 with C instead of Y at position 344); ^1 ^Interclade hybrids; ^2 ^The 'unknown Western 2' polymorphisms (light violet) were not found in direct sequencing, but are represented by a single cloned sequence and are probably present in very low amounts in this genome. Dark orange: apomorphic substitutions of Balkan species; light orange: shared substitutions of Balkan species and hybrids; green: polymorphisms shared only by Balkan interclade hybrids, but not reflected by 'pure' Balkan species ('unknown Eastern'); light violet: polymorphisms shared with the (non-Balkan) interclade hybrid accessions *H. villosum *1305 and *H. pilosum *1226/2, but not reflected by other Balkan species ('unknown Western 2'). At position 254, C occurs on the 'unknown Western 2' ribotype in *H. plumulosum *and *H. gymnocephalum*, but on the 'Eastern' ribotype in *H. olympicum *and must therefore be a parallelism. The scattered and inconclusive distribution of polymorphisms and substitutions in 'pure' Balkan species are reflected by the intra-individual patterns in cloned sequences of *H. kittanae*, their most polymorphic representative. For more details, see Additional file 2: Patterns of *ETS *recombination.

Species from the Balkans did not cluster together in phylogenetic analyses, because they mostly shared intra-individual polymorphisms rather than substitutions. These patterns were scattered across the species (Figure 4) and produced nearly all possible combinations of Balkan taxa in equally parsimonious trees (not shown). Those with only one particular nucleotide at a given polymorphic position usually showed the consensus (plesiomorphic) character state. Only four apomorphic substitutions occurred in these species; all were reflected by additive polymorphisms in other Balkan species. Most polymorphisms (21), however, were non-additive. Shared (probably ancestral) polymorphisms and geographic distribution indicate a close relationship among these species, and for the interclade hybrid *H. olympicum*, they imply a Balkan species as the most likely 'Eastern' parent, the *H. sparsum *pattern being the best match (Figure 4, Additional file 2: Patterns of *ETS *recombination).

### Non-additive, hybrid-specific variation

The *ETS *of the relict diploid *H. lucidum *showed a major ribotype corresponding to the majority of 'Western' species, but also contained a pattern of three small additional peaks and a frameshift caused by a 2 bp-indel (at positions 169, 180, 196-197, 358). The same set of intra-individual polymorphisms occurred in *H. prenanthoides *1161 (a cryptic interclade hybrid, see above), the non-consensus character states also being present in low amounts. Four interclade hybrids (*H. racemosum*, *H. sabaudum*, *H. bracteolatum*, and *H. olympicum*) showed these alternative character states in higher amounts so that they had a higher probability of being recovered by cloning. The non-consensus character states turned out to be situated on a particular physical sequence (3 out of 6 clones in *H. racemosum*, 1 out of 3 in *H. sabaudum*, 2 out of 5 in *H. bracteolatum*, and 1 out of 8 clones in *H. olympicum *plus 2 recombinant ones). We refer to this pattern as the 'unknown Western 1' ribotype. Given the diversity of origins of these six taxa as inferred from molecular markers and the large geographic distances between the sampling sites, it is unlikely that shared ancestral variation was responsible for the observed pattern in this case. More likely, the 'unknown Western 1' ribotype they have in common reflects one of the parents of all these hybrids (unsampled or extinct) while their second parent can vary. In *H. prenanthoides *1161 (see above), this involved a subsequent hybridization with a third parent (i.e. with the 'unknown Western 1').

The maternal parent of the interclade hybrids *H. pilosum *1226/2 and *H. villosum *1305 belonged to the ('Eastern') '*H. porrifolium*' clade like the 'pure' accessions of the same taxa. Their exact paternal ('Western') parent could not be identified. Both hybrid accessions shared four polymorphisms (at positions 123, 254, 332, and 357) whose non-consensus character states were situated on 'Western' *ETS *variants (designated as 'unknown Western 2', see also Additional file 2: Patterns of *ETS *recombination). These polymorphisms/ribotypes were also shared with *H. gymnocephalum *(both accessions) and *H. plumulosum *(Figure 4) which were interclade hybrids from the Balkans with unknown 'Eastern' *and *'Western' parents. One cloned sequence of *H. heterogynum*, an interclade hybrid with '*H. umbellatum*' maternal origin, showed this pattern as well. Exceptionally, these polymorphisms were not apparent in direct sequencing suggesting that this ribotype must be very rare (< 5%) in the genome of this accession and that it was probably retrieved by chance.

The latter three taxa shared another four polymorphisms (positions 162, 260, 311, 458) that were situated on 'Eastern' strands; none of these were shared with any 'pure' Balkan species (Figure 4). We refer to this pattern as the 'unknown Eastern' ribotype (see Additional file 2: Patterns of *ETS *recombination). It was confined to interclade hybrids with Balkanian distribution. Each of the 'unknown Eastern' ribotypes also contained one or two 'Balkan' polymorphisms suggesting that these variants probably arose from a Balkan species.

All three 'unknown' ribotypes occurred only in hybrids, but never as the only *ETS *variant in any other species. This is consistent with previously unidentifiable 'Western' or 'Eastern' parental subclades in all these taxa. This kind of shared variation cannot easily be considered as homoplasious, because each concerned a unique set of four intra-individual polymorphisms that were shared by particular groups of accessions and did not contradict other patterns. We therefore included all non-recombinant sequences of these variants into further phylogenetic analyses (Additional file 6: *ETS *phylogeny with ribotypes present only in hybrids). These ribotypes formed three subclades emerging from the basal 'Western' or 'Eastern' polytomies and showed similar divergences to the previously identified subclades composed of 'pure' species. The combination of direct sequencing and cloned sequences further revealed that *H. plumulosum *(diploid) comprised a total of four identifyable ribotypes: Besides the 'unknown Western 2' and the 'unknown Eastern' variant, an almost pure 'Western' sequence was found, and an ordinary 'Eastern' ribotype could also be inferred (Additional file 2: Patterns of *ETS *recombination). Likewise, *H. heterogynum *(triploid) comprised three identifyable ribotypes, but in addition had a cpDNA haplotype corresponding to a fourth lineage, the '*H. umbellatum*' group (plus two of its diagnostic substitutions in the *ETS*). A compilation of all reticulation events inferred from *ETS *patterns (including the three 'unknown' lineages) and chloroplast haplotypes is included in Additional file 5: Species/accessions, their origin, cytotype, *ETS *and cpDNA features.

### Reticulation in *Hieracium *s.str

To sum up, hybrid origin was unexpectedly inferred for a total of 29 out of 60 *Hieracium *accessions. Most of them (20) were interclade hybrids of which 17 showed complete character additivity for the two major clades; two others showed partial hybrid signatures in *ETS*, and one combined an almost exclusively 'Western' *ETS *with an 'Eastern' chloroplast haplotype. Four accessions showed evidence of multiple hybridization/introgression events. Within the 'Eastern' clade, two hybrid accessions were identified by *ETS *character additivity, and a further two were inferred from putative chloroplast capture. Within the 'Western' clade, four putative hybrids were identified by character additivity, and another species comprised an undifferentiated 'Western' and an 'unknown Western 1' *ETS *ribotype. Diploid hybrids occurred at all levels: four were interclade hybrids, two occurred within the 'Western' clade, and three within the 'Eastern' clade. One diploid comprised four different *ETS *ribotypes which suggests the presence of at least two nrDNA loci per haploid genome.

**Figure 5:**
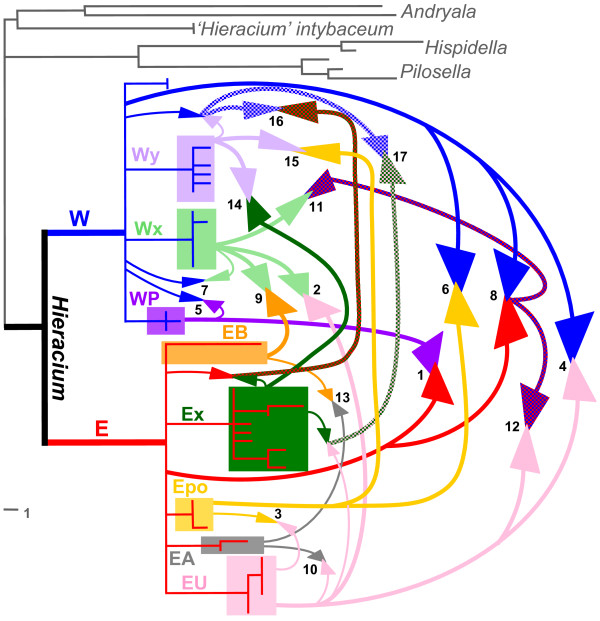
**Reticulation in *Hieracium***. A graphical summary of reticulation based on *ETS *features and (in two cases) on chloroplast capture is shown on a simplified *ETS *tree (unsupported branches collapsed) that includes also the ribotype lineages occurring only in hybrids (Wx, Wy, Ex). W - 'Western', Wx - 'unknown Western 1', Wy - 'unknown Western 2', WP - 'Pyrenean', E - 'Eastern', EB - Balkan species, EU - '*H. umbellatum*' clade, Ex - 'unknown Eastern', EA - '*H. alpinum*' lineage, Epo - '*H. porrifolium*' clade. Detailed results of this analysis are given in Additional file 6: *ETS *phylogeny with ribotypes present only in hybrids. Small arrows/thin lines represent hybridizations within each clade, larger arrowheads/bold lines indicate involvement of both major clades. If no particular subclade could be inferred for a given combination, arrows start at the polytomies of the 'Western' or 'Eastern' clades. Multiple hybridizations are indicated by patterned arrows. They reflect particular ribotype combinations, not any order of subsequent hybridizations. Numbers near the arrowheads refer to particular hybrid accessions: 1 - *H. amplexicaule*, *H. gouani*; 2 - *H. bracteolatum*, *H. racemosum*, *H. sabaudum*; 3 - *H. bupleuroides *1033, 4 - *H. caesium*, *H. lachenalii*, *H. laevigatum*; 5 - *H. candidum*, *H. cerinthoides*, *H. cordifolium*, *H. gymnocerinthe*; 6 - *H. glaucum*; 7 - *H. lucidum*; 8 - *H. mixtum*, *H. prenanthoides *1252, 9 - *H. olympicum*; 10 - *H. pojoritense*; 11 - *H. prenanthoides *1161, 12 - *H. prenanthoides *1187, 13 - *H. sparsum *(2); 14 - *H. gymnocephalum *(2), 15 - *H. pilosum *1226/2, *H. villosum *1305, 16 - *H. plumulosum*; 17 - *H. heterogynum*.

Altogether, at least 17 different combinations of parental lineages were found in the accessions with hybrid origin. They included three ribotypes that occurred only in hybrids. A graphical summary of the inferred intra- and interclade reticulation patterns is provided in Figure 5. A more detailed assessment and discussion of the origin of each species/accession, including some ecogeographic, morphological, and floristic information are given in Additional file 1: Origins of individual accessions.

## Discussion

### NrDNA patterns revealed by direct sequencing and cloning

In our experience ([54], Krak et al., unpubl. data) and that of other labs [32,55], the relative ratios of parental sequence types in PCR amplicons are usually reproducible. Also, in the present study, relative proportions of cloned sequences - generated from separately amplified PCR products - roughly corresponded to relative amounts of superimposed peaks observed in direct sequencing. Furthermore, relative amounts of parental *ETS *alleles in interclade hybrids could be biased towards the 'Eastern' *or *the 'Western' variants (Additional file 5: Species/accessions, their origin, cytotype, *ETS *and cpDNA features) and corresponded to total genome size of the respective accessions [46]. This suggests that backcrossing or unequal genome composition (e.g., diploid and haploid gametes contributing to a triploid) generally had a stronger influence on different *ETS *ratios in hybrids than concerted evolution. Reproducibility of relative amounts of amplified parental variants, biased ratios occurring in either direction, and matching genome sizes argue against the confounding effects of PCR selection or drift [56] in our data.

The reliability of direct sequencing is also highlighted by a comparison of the strength of particular signals at polymorphic positions in direct sequencing with recombination patterns in cloned sequences. Direct sequencing was usually superior in cases of heavily biased representation of different ribotypes (for examples, see Additional file 2: Patterns of *ETS *recombination), because it can sample variation across all nrDNA loci present in the genome. Rare alleles - contributing less than about 5% of the total signal in electropherograms in our case - are equally difficult to identify by cloning [22,32], especially if there is no prior information that such variation exists. While we cannot exclude that cloning of additional accessions might have revealed still further ribotypes, the example of *H. kittanae *- a 'pure' diploid species with the highest number of polymorphisms (15) - which reflected the inconclusive patterns of Balkan species (Figure 4) by an equally inconclusive pattern at the intra-individual level (Additional file 2: Patterns of *ETS *recombination) raises doubts that further extensive cloning would have retrieved much new information. Besides, in cloned sequences, polymerase errors, intragenomic and/or PCR recombination, single substitutions on the 'wrong' strand or hybrid-specific sequences, the latter probably resulting from gene conversion, have to be disentangled from 'original' or major parental ribotypes (see Methods and Additional file 2: Patterns of *ETS *recombination). This becomes an impossible task if these variants do not differ by a sufficiently high number of diagnostic characters. On the other hand, cloning was necessary to resolve or confirm sequence reads affected by multiple indels and to attribute the derived character states of shared informative polymorphisms to particular physical sequences (e.g., for the 'unknown' ribotypes).

### Intra-individual polymorphism in the *ETS*

The earlier view that nrDNA repeats are generally homogeneous within a species [22] has led to the widespread assumption that intra-individual polymorphism of nrDNA is the exception rather than the rule [57]. More recently however, researchers have become increasingly aware of the problems associated with the complex and often unpredictable behavior of this multicopy marker [9]. By 2003, Bailey et al. [58] can already cite 22 reports of intra-individual polymorphism. Meanwhile, this is thought to be a common phenomenon in plants [59], and a certain degree of intra-individual polymorphism can usually be found whenever it is looked for, though it is still widely ignored [60].

The most frequently observed reasons for *prominent *intra-individual polymorphism of nrDNA are hybrid/allopolyploid origin [27,28,30,32,33,61,62] or the occurrence of pseudogenes [63-67]. We consider the latter possibility as unlikely for *Hieracium *because overall sequence variation was very low, the GC content of all accessions fell within a very narrow range (50.4-51.9%), and long indels were missing. Other criteria for the recognition of pseudogenes are not easily applicable, because *ETS *- unlike the *ITS *region - does not comprise conserved genes, and comparative data on secondary structure are also not available. Reticulation as one source of the extensive intra-individual polymorphism in *Hieracium *is supported by the fact that 17 different combinations of ribotypes accounted for a considerable number of the sequence polymorphisms observed in individual accessions; most of them involving 'Eastern' *and *'Western' variants. All accessions with more or less homogeneous *ETS *fell into one or the other clade (Figure 5). However, one could also argue that multiple paralogous ribotypes within individual genomes might reflect ancestral polymorphism and lineage sorting [68] rather than reticulation. This would require that the ancestral population of the whole subgenus already comprised all ribotypes as divergent *ETS *alleles or paralogs. Accessions with composite genomes could then be understood as heterozygous individuals derived from various combinations of original *ETS *ribotypes already present in the ancestral population. The 'pure' species would then be homozygous for one of the divergent *ETS *ribotypes, have suffered locus loss, or their ribotypes became homogenized towards particular variants by concerted evolution. The phylogeny (Figure 2) would in that case represent a gene tree rather than a species tree. However, the geographic pattern along with the divergent genome sizes observed for the major clades, the general congruence of cpDNA haplotypes with *ETS *subclades/species groups (Additional file 5: Species/accessions, their origin, cytotype, *ETS *and cpDNA features), and the correspondence of ecological preferences or morphological similarities of species belonging to particular (e.g., '*H. porrifolium*', '*H. umbellatum*') subclades strongly argue in favor of true species relationships. In contrast, lineage sorting is expected to result in randomly distributed ribotypes following divergence from a polymorphic ancestor. Only the incongruent patterns observed in Balkan species (Figure 4) show features that are best explained by ancestral polymorphism and lineage sorting (see Results). For most of our data, however, this is not the most parsimonious explanation. Instead, we consider hybridization among members of divergent lineages to be a more likely interpretation, at least for the additive patterns.

In many cases, a considerable number of intra-individual polymorphisms were superimposed on the variation due to additivity. While shared informative polymorphisms contained additional phylogenetic signal (see Results), most of the non-additive variation was unique/accession-specific (Figure 1), i.e., phylogenetically uninformative. These polymorphisms were either randomly scattered on different physical strands or tended to have accumulated in a particular ribotype (e.g., 'Eastern' or 'Western', see Additional file 2: Patterns of *ETS *recombination). This is consistent with the assumption that they resulted from mutations occurring on different nrDNA repeats and had spread in the genome to some degree - in order to be detected at all in direct sequencing, they have to reach a threshold of at least 5% of all copies - but failed to be completely homogenized by concerted evolution (similar to retained parental variants in most hybrids). The large amount of unique or apparently randomly distributed (homoplasious) intra-individual variation (Figure 1) may indicate partial saturation with intra-individual polymorphisms suggesting slow concerted evolution relative to mutation rates. The occurrence of two or even three different kinds of polymorphism at 35 positions points in the same direction. At one of these positions (254), C as a derived character state evidently arose twice independently; once as part of the 'unknown Western 2' ribotype, and once on the 'Eastern' alleles of Balkan species (Figure 4, Additional file 2: Patterns of *ETS *recombination).

Due to the complex patterns and abundance of intra-individual variation, prior to phylogenetic analysis of the *ETS *dataset, noise had to be distinguished from meaningful variation, and reticulation had to be identified as such, because hybrids are known to confound phylogenetic analysis when they remain undetected [69,70]. Almost half of all accessions analyzed showed evidence of reticulation. Even restricting the analyses to diploids was not an option in this case, because diploids were equally polymorphic on average and also included interclade hybrid accessions. In fact, the highest number of polymorphisms occurring in any accession (37) was found in a diploid (a hybrid containing four divergent ribotypes plus many unique polymorphisms) while the lowest numbers (1 or 2 polymorphisms) occurred in some triploids and tetraploids of presumed autopolyploid origin. Although a number of programs are currently available that can deal with intra-individual variation and reticulation [59,71,72], no algorithm can possibly distinguish between meaningful and homoplasious patterns if they occur at the same aligned positions, or identify hybridization from partial additivity or from novel hybrid sequences (e.g., *H. prenanthoides*, *H. mixtum*). Similarly, in a recent study on *Fagus *where a high level of intra-individual polymorphism in the *ITS *region was detected [73], only detailed visual investigation of the variability patterns allowed the inference of the basic phylogenetic patterns. Also, in a study of sexual *Erigeron *species that showed frequent intra-individual polymorphism in *ITS *and *ETS *sequences, a fair degree of handwork had to be done to identify diagnostic/additive patterns, to distinguish informative from uninformative variation, and to detect recombination in cloned sequences [44]. However, reticulation explained most of the observed variation in that case - in contrast to our dataset where the majority of intra-individual variation was uninformative or even misleading.

### Basic patterns of *Hieracium *evolution

The basal split into a 'Western' and an 'Eastern' clade suggested by *ETS *was not predicted by morphology nor suggested in any taxonomic treatment. In the light of our data, we suggest several reasons for that failure: (i) both clades include species (groups) with unique morphology, (ii) both comprise species with large or Central European distribution areas, (iii) the large number of interclade hybrids has confounded the picture, and (iv) several morphologically defined taxa had multiple origins and are probably inadequately circumscribed. Dating of this basal divergence is not feasible because of a lack of fossils; fossil pollen cannot be attributed to particular genera in the Asteraceae subfamily Lactuceae [74]. However, there are some indications that suggest a Quaternary timescale, at least for speciation within both major clades (see below).

Low overall *ETS *sequence divergence indicates that, despite rather high morphological diversity even within each clade/subclade, speciation was not accompanied by significant molecular differentiation. Similar observations have been made in other plant genera [75,76]. *ETS *sequence divergence in many other Asteraceae genera is considerably greater [12,77-80]. Exceptions are genera that have a restricted geographic range, were sampled only from a restricted area, underwent rapid recent speciation, experienced population bottlenecks, or a combination of these [12,81,82]. The first two reasons do not apply here as our sampling covered the entire, rather large distribution area of *Hieracium *(North America to Siberia, Scandinavia to the Mediterranean). An indication of rapid recent speciation is the extremely low level of *ITS *variation in *Hieracium *s.str. ([13], and unpublished data). While taxonomic levels are not strictly comparable between groups, even *ITS *sequence divergence, although often markedly lower than *ETS *variation, has proved useful for the inference of plant relationships at species and genus level in numerous studies. This might suggest a rather recent divergence even of the two major lineages identified by the *ETS *region. An especially rapid diversification of lineages could be reflected by the basal polytomies of both major clades. The coincidence of the distribution of many diploid and endemic taxa with known glacial refuge areas [83,84] may indicate that these are remnants of originally much larger populations or higher species diversity. The Quaternary in Europe was characterized by many range expansions/contractions related to climatic oscillations which could have resulted in repeated population bottlenecks [85]; this may help to explain the low genetic diversity in our data. After the retreat of the Pleistocene ice sheets, rapid speciation within the 'Eastern' and 'Western' lineage may have been facilitated by the availability of vast areas open to colonization [86]. Subsequently, sympatric speciation by hybridization among these previously separated lineages - accompanied or followed by polyploidization or introgression at the diploid level - may have provided a quick way to adapt to new ecological conditions and to invade new habitats. The obvious correspondence of the distribution areas of many non-hybrid endemics and diploids with known glacial refuge areas and the abundance of interclade hybrids suggest extensive reticulation after the two lineages came into secondary contact. This is in accordance with Stebbins' secondary contact hypothesis [86], which is corroborated by numerous case studies (e.g., [28,87,88]), albeit mostly at the intraspecific level. Above species level, a similar east-west differentiation that may be related to survival in different glacial refuge areas was found in *Hieracium *subgenus *Pilosella *although in that case it mainly concerns divergent chloroplast haplotypes [89].

In the wake of polyploidization, the onset of apomictic reproduction may also be linked to climatic change during or after the Ice Ages [89]. Most diploids are nowadays confined to the southern part of the distributional range of the genus from where the regions further to the north were later re-colonized, which is also a common pattern in other apomictic plants [90]. The largest sequence divergence was found among and within diploids. There is no indication that polyploidy as such has added much to the overall genetic variation. However, polyploidization, frequent hybrid origin, and fixation and spread of genotypes by apomixis have generated the huge number of taxa which makes *Hieracium *one of the largest plant genera, even if a broad species concept is adopted. The large number of accessions with interclade hybrid origin shows that members of the two major clades were not yet reproductively isolated when the reticulation occurred despite their differences in genome size which are reflected by the genome sizes of their hybrids [46].

### Indications for ancient hybridization

Even taxa having composite genomes according to our data have unique morphology - reflecting Zahn's [3] definition of a 'basic' species - which is why their hybrid identity had not been assumed before. However, contemporary hybridization is thought not to play a large role in *Hieracium *s.str. [37]. In the following, we discuss several lines of evidence for ancient hybridization with a special focus on diploids with hybrid origin.

#### Geography

Many diploid *Hieracium *species are not sympatric and/or are ecologically isolated. Diploid hybridogenous accessions (or species) that are particularly unlikely to have formed recently are *H. gouani*, *H. gymnocephalum *1215, *H. lucidum*, *H. plumulosum*, *H. pojoritense*, *H. prenanthoides *1252, and *H. sparsum*. All occur in areas that have never been glaciated, or only partly. Some of these (*H. lucidum*, diploid *H. prenanthoides*) have particular relict character, i.e., their populations are most likely remnants of a previously much larger distribution, or their localities are typical refugia (*H. gouani*, *H. pojoritense*, *H. plumulosum*). In case of *H. lucidum*, pollen producing polyploids like *H. crinitum *(treated as a subspecies of *H. racemosum *by Zahn [3]), could have played a role in past hybridization events [91]. Nowadays, no population of any other *Hieracium *species occurs in the neighborhood of *H. lucidum*. Thus, recent hybridization events among diploids are highly unlikely.

#### Molecular patterns

*ETS *signatures of diploid accessions of *H. prenanthoides *and *H. gymnocephalum *were also found in polyploids of the same species indicating that these diploids with hybrid origin have given rise to widespread polyploids assigned to the same taxon. *Hieracium gymnocephalum *is a morphologically very variable and mostly triploid aggregate - our accession 1215 represents the first report of a diploid in this species [46] - whose variation is thought to be caused by past hybridization or introgression [92]. According to *ETS*, *H. sparsum *occurs at an unresolved position within the 'Eastern' clade, and two accessions from different localities were nearly identical in their molecular features. Thus, genetically, *H. sparsum *s.str. behaves like a 'good species' which is in accordance with morphological and floristic observations. While we analyzed mostly diploid accessions of these taxa, *H. sparsum *s.l., *H. prenanthoides *s.l., and *H. plumulosum *(*H. waldsteinii *s.l.) are predominantly polyploid species and are supposed to have been involved in the formation of many 'intermediate' taxa - some of them widespread -, which is suggestive of extensive ancient hybridization, particularly of the diploid progenitors with hybrid origin.

#### Reproduction

All diploid accessions analyzed here are sexuals with normal seed production [46] while a rare recent natural hybrid was sterile [37]. Experimental hybridization of diploids also resulted in mainly or completely seed-sterile hybrid progeny [93]. Whilst we did not analyze the breeding system of *H. lucidum *- because of its critically endangered status, no living plants were collected - isozyme analyses showed some variation, which is in accordance with sexual reproduction and also with a small population size over a rather long period of time [91]. Apomicts of *Hieracium *have very variable male fertility, ranging from almost complete sterility to normal pollen fertility [52,94,95]. Triploid apomicts, which represent the vast majority of *Hieracium *species, can produce haploid pollen and could therefore fertilize diploid accessions (resulting in diploid hybrid progeny). This would be the only way to overcome the geographic isolation of most recent diploids and to produce significant numbers of hybrids nowadays. However, pollen from other species can lead to a breakdown of self-incompatibility resulting in autogamy. This so-called mentor effect has been demonstrated to be especially strong for pollen from apomictic triploids in *Hieracium *s.str. [95]. Nowadays, autogamy enforced by contact with foreign pollen is a very efficient way to avoid hybridization.

Finally, since most of these *Hieracium *species had been described prior to 1900, many by Linnaeus himself, it is unlikely that hybridizations that gave rise to 'basic' species occurred within the last few hundred years.

Thus, the unanticipated hybridizations involved in the formation of many of the 'basic' species may actually be rather ancient, and may even date back to the early Holocene (see above). This would also provide a reasonable time frame for the formation and spread of the large number of 'intermediate' *Hieracium *species, whose morphology suggests a hybrid origin involving two or more of the 'basic' species.

### Evidence for the contribution of variation from extinct taxa

Several lines of evidence suggest a contribution from either unsampled taxa or from extinct lineages that have left molecular traces in the accessions analyzed. We consider contribution from extinct forms more likely, for the following reasons.

The 'unknown Western 1' ribotype, occurring in six hybrid species, at least four of which have different origins, was not found as the only *ETS *variant in any existing species. The respective accessions were collected from sites as distant as Sicily, Poland and the Balkans. The 'unknown Western 1' ribotype may have originated from a lineage/species that was either once widespread, given the collection sites of the hybrid accessions in which it was found, or its genome has spread along with apomictic polyploids after hybridizations in a Western European region. Similarly, the 'Western' parent of six interclade hybrids represented by five different species corresponded to the 'unknown Western 2' lineage. This ribotype also has a large geographic distribution according to the sampling sites (France, Southeastern Alps, Southern Balkans). A further ribotype present only in hybrids ('unknown Eastern') occurred in four accessions of three species and was restricted to a particular part of the Balkans (Albania, Montenegro).

Chloroplast DNA haplotypes of several hybridogenous accessions (*H. plumulosum*, *H. olympicum/H. gymnocephalum*, *H. mixtum*) were unique and fairly divergent from others given the low overall variation of this marker (Figure 3), i.e., a candidate maternal parent is missing. This could be an indication that more diploids existed than are known today, since apomictic polyploids can only act as pollen donors and therefore cannot contribute to cpDNA diversity. Even allowing for the accumulation of mutations in the cpDNA after ancient hybridizations does not explain why the majority of hybrid chloroplast haplotypes were identical to one of the parental groups inferred from the *ETS*. Almost identical cpDNA haplotypes occurred in *H. olympicum *('unknown Western 1'/Balkan) and *H. gymnocephalum *('unknown Western 2'/'unknown Eastern'). Given their genomic composition according to *ETS*, they seem to share a maternal progenitor that does not correspond to any of the identified ribotype lineages.

Whilst insufficient sampling can never be excluded, it is unclear what the identity of the species providing the missing variation could possibly be or where they should occur. The only 'basic' species not included in our study were *H. fuscocinereum*, a Northern European polyploid species with some similarities to *H. murorum*; *H. schmalhausenianum*, a Caucasian endemic with unknown ploidy; and *H. laniferum*, a diploid occurring in parts of Spain. The latter is the only candidate that could theoretically consist exclusively of one of the 'unknown Western' *ETS *ribotypes, but it could well be of hybrid origin itself, like half the other 'basic' species or be genetically indistinguishable from the majority of 'pure' species of the 'Western' clade. In any case, these three unsampled taxa certainly cannot account for all the missing variation, i.e., the second 'unknown Western' and the 'unknown Eastern' ribotype, and at least three divergent hybrid chloroplast haplotypes (even if one of them corresponds to an 'unknown' *ETS *lineage). There is also no explanation why 20% (12 out of 60) of the accessions collected from distant geographic sites should comprise these ribotypes, but the ancestral species have always been missed by chance - for the other subclades whose ribotypes also occurred in hybrids, 'pure' candidate parental species were present. Unsampled intraspecific variation as a potential source of the missing variation is also unlikely. Taxa with more than one accession analyzed showed either (i) the same patterns, independent of ploidy, 'pure' species status, or inferred hybrid origin, or (ii) one accession represented a 'pure' species while others were hybrids involving that species and other taxa. Also, the majority of *Hieracium *populations are polyploid and, thus, are unlikely as predecessors. We therefore consider it more likely that extinct species have left their molecular traces in the genomes of taxa with rather ancient hybrid origin.

Species number also argues for the existence of a large range of extinct diversity. Out of the 46 'basic' species analyzed, which were initially considered as the main evolutionary units, only ten diploid species did *not *show molecular evidence of hybrid origin. Theoretically, ten parental species can produce 45 different hybrid combinations (or 90, if reciprocal crosses are considered as different hybrids). However, at least 32 diploids would be needed to explain the species numbers in *Hieracium*, if only macrospecies were taken into account (500). As it is very unlikely that each possible parental combination would be realized in nature, the original number of diploids should probably be much higher.

Morphological indication of potential hybrid origin involving particular recent species is also missing from the analyzed taxa, a fact that is reflected by their treatment as 'basic' species which by definition do not show evidence of character combinations of other species. In contrast, for the multitude of 'intermediate' taxa not analyzed here, morphology does suggest putative parents. Experimental hybridization of several diploid *Hieracium *species showed that hybrid progeny of the same cross can exhibit a large range of morphological variation, but generally, hybrids were either intermediate between their parents or more similar to the maternal one [93]. Thus, hybrid origin from extant parents should usually be detectable. Taken together with the relict character of most diploids, this points to a greater ancestral species diversity now reduced by extinctions. This could - among several other factors - contribute to explaining some of the taxonomic problems in *Hieracium*: As morphological characters of extinct species are naturally unknown, hybrid origin of extant taxa can easily go undetected due to the lack of one or both parents. Even hybridization of the same two parents may result in strikingly dissimilar phenotypes, depending on the particular contribution of gametes. One of the best known examples is the hybrid origin of three different sunflower species from the same parental species combination [96]. However, as long as the parental species and their morphological diversity are known, correct determination of polymorphic hybrids by an expert taxonomist is still feasible [36,97]. However, if only one parent is extinct, even morphological identification of the same hybrid taxon may become impossible.

Our findings add to an increasing number of studies in which the contribution of extinct parent(s) was inferred from molecular data [30,98-103].

## Conclusion

The initial aim of this study was to disentangle relationships and species origins of the taxonomically highly complex *Hieracium *s.str. for the first time using molecular markers. The particular features of the *ETS *dataset, characterized by abundant intra-individual variation, most of which was uninformative and some even misleading, required a very detailed inspection of the nature of this variation in order to retrieve the phylogenetic signal and to identify the unexpected hybrid origins of roughly half of all accessions analyzed (Figure 5). Whether the complex data structure is the result of the particular history and extensive apomixis in *Hieracium*, or whether similar molecular patterns will be found in other groups, remains to be seen. The approach used here for the analysis of intra-individual polymorphism might act as a model for the study of other agamic complexes and other similarly challenging patterns of molecular data.

## Authors' contributions

JF conceived of the study, did the data analyses and interpretations, and wrote the manuscript. KK was responsible for molecular data acquisition, provided part of the Methods section and critically revised the molecular data files and the manuscript. JC was responsible for collection and determination of the plants and provided chromosome counts, genome size data, information on breeding systems, and taxonomic background for the paper. All authors read and approved the final manuscript.

## Supplementary Material

Additional file 1 **Origins of individual accessions**. A detailed assessment of the origin of each species/accession based on *ETS *features (phylogeny, shared polymorphisms), cpDNA haplotype, ploidy and genome size is given. The file also includes some ecogeographic, morphological and floristic information and a table containing source information for all accessions including the outgroup. Click here for file

Additional file 2 **Patterns of *ETS *recombination**. Cloned sequences of nine interclade hybrid accessions (*H. olympicum*, *H. mixtum*, *H. prenanthoides *1187, *H. caesium*, *H. pilosum *1226/2, *H. villosum *1305, *H. gymnocephalum *1215, *H. heterogynum*, *H. plumulosum*) and *H. kittanae*, the non-hybrid accession with the highest number of intra-individual polymorphisms, are shown. Cloned sequences are compared with intra-individual polymorphisms and dominant character states revealed by direct sequencing. All cloned accessions with recombinant sequences are included. Patterns of gene conversion and the distribution of accession-specific polymorphisms across the cloned sequences can be traced. Color coding of diagnostic character states as in Figure 5. Click here for file

Additional file 3 **Summary of intra-individual polymorphisms**. All intra-individual polymorphisms are listed according to their position in the alignment along with the accessions in which they occur, discriminating additive, unique, and shared variation. Among the shared polymorphisms, homoplasious and informative ones are identified according to specified criteria. Click here for file

Additional file 4 **Alignment of *Hieracium ETS *sequences**. A FASTA file is given comprising two sequences for each accession (i) one with all polymorphic sites included (lower case letters indicate weak [i.e., second peak small] or indel polymorphisms): the sequence name is composed of the abbreviated species name and the number of the accession/plant, and (ii) one resolved for the major sequence in case of skewed ratios: same label, but preceded by an 'M'. Additionally, cloned sequences are given for the respective accessions: same label, but preceded by the clone number. The positions in the alignment correspond to Table 1, Figure 4, and Additional files 2 and 3. Click here for file

Additional file 5 **Species/accessions, their origin, cytotype, *ETS *and cpDNA features**. This table summarizes the information about individual accessions. Click here for file

Additional file 6 ***ETS *phylogeny with ribotypes present only in hybrids**. The file contains the phylogenetic analyses on which Figure 5 is based. One out of 1402 equally parsimonious trees is shown (1752 steps, ri = 0.952, ci = 0.982; 182 variable characters of which 95 were parsimony informative) with bootstrap support indicated above the branches. The strict consensus tree topology corresponds to the branches with support values. Bootstrap values for ML and posterior probabilities for Bayesian analyses are given below the branches. Diploid *Hieracium *species are indicated in boldface. All lineages comprise diploids (the 'unknown Western 1' ribotype also occurs in low amounts in diploid *H. lucidum *which was not cloned). The Wx, Wy, and Ex lineages are represented by all non-recombinant clones of these ribotypes. Identical clones of the same accession were included only once (see also Additional file 2: Patterns of *ETS *recombination). The four hybrid accessions still maintained in Figure 2 were excluded, and also *H. lachenalii *and *H. sparsum *because of inferred chloroplast capture. Figure 5 uses the basic structure of this tree on which all inferred reticulation events were mapped. Click here for file
