# Supplementary material for: Intra-individual polymorphism in diploid and apomictic polyploid hawkweeds (Hieracium, Lactuceae, Asteraceae): disentangling phylogenetic signal, reticulation, and noise
Source: BMC Evol Biol. 2009 Sep 22;9:239. doi: 10.1186/1471-2148-9-239 (PMC2759941; doi:10.1186/1471-2148-9-239)
Supplement: Additional file 1 — Origins of individual accessions. A detailed assessment of the origin of each species/accession based on ETS features (phylogeny, shared polymorphisms), cpDNA haplotype, ploidy and genome size is given. The file also includes some ecogeographic, morphological and floristic information and a table containing source information for all accessions including the outgroup. [file 1471-2148-9-239-S1.PDF]

Additional file 1: Origins of individual accessions

In the following, we concentrate on species as taxonomic entities. Each was represented by only one or a few samples, and therefore, the discussion of the molecular data applies only to the particular accessions analyzed. In some cases, we could show particular taxa to be inhomogeneous entities – which is interesting in itself – and this may apply to other species as well if a larger number of accessions will be investigated. Nevertheless, a preliminary assessment of accessions in relation to the taxon as a whole provides useful insights and generates working hypotheses that can guide future research strategies and sampling schemes.

Source information for all accessions and outgroup taxa is given in a table at the end of this file.

***H. humile* Jacq.**

The species is morphologically more or less uniform and has its major distribution in the Western Alps. Two accessions, a triploid and a tetraploid one, were analyzed. *ETS* placed both accessions into the 'Western' clade. Together with one accession of *H. pilosum*, *H. humile* was the species with the lowest number of polymorphic sites (1–2) despite the polyploidy of the analyzed accessions. It is possibly an old species; morphologically, it cannot be derived from any recent species. The chloroplast DNA of both accessions was identical to that of other 'Western' clade species. The low amount of intra-individual polymorphism suggests autopolyploid origin from extinct diploid accessions of the same taxon, because diploid populations are unknown.

***H. tomentosum* L.**

This species occurs in the Western Alps and also belonged to the 'Western' clade. A diploid accession shared one polymorphic site with the majority of 'Western' species, reflecting a substitution characteristic for the 'Pyrenean' lineage. A similar structure of the indumentum shared between *H. tomentosum* and some Pyrenean species could also be indicative of a loose relation. Most other polymorphisms were unique. Its cpDNA haplotype was 'Western'.

***H. murorum* L.**

This species is distributed all over Europe. Despite its being one of the morphologically most polymorphic taxa in *Hieracium*, intraspecific differences are still much smaller than, for example, in *H. prenanthoides* (see below). Although this is a polyploid taxon, the analyzed triploid accession showed a relatively low number of *ETS* polymorphisms suggesting autopolyploid origin. According to *ETS* and cpDNA, it belonged to the 'Western' clade. Analysis of additional accessions might reveal genetic heterogeneity of this species.

***H. bifidum* Kit.**

This is a Central and North European, predominately mountainous calciphilous species with morphological similarities to *H. murorum* and *H. stelligerum*. The analyzed triploid accession from the Slovakian mountains had a moderate amount of polymorphic sites and was of western origin according to nuclear and chloroplast DNA.

***H. schmidtii* Tausch**

The distribution of this Mediterranean-Atlantic species extends in Western Europe far to the north; the further to the east, the more southern the distribution becomes, reaching Lebanon. The analyzed Czech accession (triploid) belonged to the 'Western' clade according to *ETS*, cpDNA, and DNA content. Intra-individual polymorphisms were either unique or homoplasious, i.e., shared with different species without any apparent pattern. The species has special ecological demands (canyon-like valleys, rock-exposed if local humidity is high) and a fragmented distribution area.

***H. pictum* Pers.**

This polyploid species occurs in the Western Alps (Southwestern Switzerland, France, Northwestern Italy), rarely also in Central Italy. It is morphologically more or less uniform, and resembles *H. bifidum* and an intermediate species between *H. bifidum* and *H. schmidtii* (*H. hypochoeroides*, syn. *H. wiesbaurianum*). *ETS*

and cpDNA of both triploid accessions corresponded to a ‘Western’ origin. Despite being polyploid, only a small number of polymorphic sites (1 and 4, respectively) occurred which differed between the accessions. Autopolyploid origin from extinct diploids of the same taxon is probable.

***H. stelligerum* Froel.**
It is morphologically rather uniform, only indumentum and leaf shape are highly variable. Only ca 15 populations in a small area of Southern France are known, highlighting the relict character of this diploid species. It co-occurs with *H. schmidtii*, *H. murorum*, and *H. bifidum* whose analyzed accessions also belonged to the ‘Western’ clade. Its ecology is similar to *H. bifidum* with which it shared two out of three polymorphic sites in *ETS* and a ‘Western’ cpDNA haplotype. The taxon could be ancestral to polyploid *H. bifidum*.

***H. transylvanicum* Heuff.**
This is a frequent species in the Eastern and Southern Carpathians and also occurs in the Eastern Alps and the Northern Balkans. It is more or less uniform morphologically, some plasticity concerns only the growth form. Despite its eastern distribution, *ETS* sequences placed both diploid accessions into the ‘Western’ clade. The cpDNA was unique; the DNA content was far above the usual values for ‘Western’ species. Accessions 1077 and tra.Boa differed in their polymorphic sites; tra.Boa shared a single one with several hybrids at a homoplasious position, 1077 shared one with some species from the ‘Western’ clade, one with Pyrenean taxa, and two reflected substitutions of accession tra.Boa. Two alternative scenarios for the origin of this species are proposed: 1) The species has an originally eastern origin as suggested by its current distribution and DNA content and was introgressed a long time ago by species from the ‘Western’ clade some of which are or were widespread. *ETS* sequences then became completely homogenized towards the ‘Western’ type. In this case, the introgression by ‘Western’ species should be rather ancient given complete homogenization of *ETS* towards this type plus additional unique substitutions. 2) The species originated in Western Europe, spread towards the east, the original populations became extinct (probably during the Ice Ages), and only the eastern populations survived in eastern glacial refugia like the Carpathian basin. In this case, the high DNA content must have other reasons than reflecting phylogenetic signal. Shared polymorphisms with different ‘Western’ clade species in accession 1077 could then be interpreted as retained ancestral polymorphism and, together with a unique cpDNA, imply an old, truely western origin. The first scenario may be more probable, because *H. transylvanicum* is rather frequent in Eastern Europe, does not have any relict populations in Western Europe, and apparently did not leave any traces in intermediate apomictic taxa.

***H. lucidum* Guss.**
This critically endangered species (*sensu stricto*) is only known from a single relict population in Northwestern Sicily. The diploid accession belonged to the ‘Western’ clade and showed a high level of polymorphic sites; more than half of them were unique. Four intra-individual polymorphisms were indicative of the ‘unknown Western 1’ ribotype which was present in low amounts. The chloroplast haplotype was identical to that of two accessions of *H. prenanthoides* (1252 and 1187). Accession 1252 was diploid and restricted to the Southwestern Alps supporting a western origin also for *H. lucidum*. The data are concordant with ancient hybridization between a ‘Western’ species (but not extant *H. prenanthoides*) and an unsampled or extinct species of western origin. Morphologically, *H. lucidum* has some similarity with *H. racemosum* which could be caused by their sharing an ‘unknown Western 1’ genome. Also, *H. crinitum*, a polyploid subspecies of *H. racemosum*, is rather frequent in Sicily and may have introgressed *H. lucidum* in former times. Nowadays, the only remaining population of *H. lucidum* is geographically isolated from other *Hieracium* species.

***H. lawsonii* Vill.**
This species is from the Pyrenees and the Western Alps. A triploid plant shared most of its few polymorphic sites with other Pyrenean species and, according to *ETS* and cpDNA, belonged to the ‘Pyrenean’ subclade.

***H. ramondii* Griseb.**
A triploid of this Pyrenean taxon shared two out of three polymorphisms with other Pyrenean species. It also belonged to the ‘Pyrenean’ subclade, but had another ‘Pyrenean’ cpDNA haplotype than *H. lawsonii*.

***H. recoderi* De Retz**

This Pyrenean species shared four polymorphic sites with other species from the same clade, three of them mostly with other Pyrenean taxa. *ETS* attributed it to the 'Pyrenean' clade of which it was the only diploid representative; its cpDNA haplotype was the same as in *H. ramondii*.

***H. cordifolium* Lapeyr.**

This diploid Pyrenean species shared a few polymorphic sites with other 'Pyrenean' and 'Western' clade taxa, other polymorphisms were unique. It is probably a hybrid between 'Pyrenean' and undifferentiated 'Western' taxa. Its cpDNA haplotype was derived from one of the 'Pyrenean' haplotypes.

***H. gymnocerinthe* Arv.-Touv. & G. Gaut.**

Like most other Pyrenean species, the analyzed triploid accession shared polymorphic sites with 'Pyrenean' and some other 'Western' clade taxa, one site with all accessions of *H. prenanthoides*. Two polymorphisms at homoplasious positions occurred also in different 'Eastern' species. The cpDNA haplotype was one of the 'Pyrenean' variants. We consider it as a hybrid between the 'Pyrenean' lineage and other 'Western' taxa.

***H. candidum* Scheele**

Like the previous species, the triploid accession shared polymorphisms with other 'Pyrenean' taxa, some with basal 'Western' clade species, a few probably homoplasious ones also with different 'Eastern' taxa. One polymorphic site was exclusively shared with *H. amplexicaule*. Its cpDNA haplotype was one of the two 'Pyrenean' variants. Similar hybrid origin as for the preceding species is suggested.

***H. cerinthoides* L.**

The degree of morphological polymorphism of this Pyrenean species is comparable with that of other Pyrenean taxa. All six polymorphic sites in the *ETS* of the triploid accession were shared with 'Western' clade taxa, predominantly with other Pyrenean species. Its cpDNA haplotype corresponded to one of the 'Pyrenean' variants. The accession had most likely hybrid origin involving a 'Western' clade taxon and one belonging to the 'Pyrenean' lineage.

***H. gouani* Arv.-Touv.**

This species occurs at the foothills of the Pyrenees in refugial localities and has relict character. It belongs to the *H. cerinthoides* group. The only co-occurring *Hieracium* species is *H. sabaudum*. The analyzed diploid accession was a hybrid between the 'Eastern' and 'Western' clade as it showed additive sites at all positions differing between the clades. In addition, it shared six polymorphisms with different Pyrenean species indicating that a taxon from the 'Pyrenean' subclade hybridized with an 'Eastern' clade species (but probably not with *H. sabaudum*, see below). No particular 'Eastern' clade taxon could be identified. The 'Western' sequence type was by far dominating (about 90% across the sites) which fits to its low genome size and its chloroplast DNA of Pyrenean origin.

***H. pilosum* Schleich. ex Froel.**

This is a morphologically variable Central European high mountain taxon. In morphology and distribution, it resembles *H. villosum*. Of two triploid accessions from the same locality in the Slovenian Alps, one had 'Eastern' origin, the other was a hybrid between the two major clades. The 'pure' species had only a single polymorphic site which it shared with other species of the same 'Eastern' ('*H. porrifolium*') subclade. The hybrid accession showed strong additivity of positions differing between the major clades. In addition, six polymorphisms were shared with different species of the '*H. porrifolium*' subclade, mostly with *H. villosum*. Four further polymorphisms – consistently shared with several other hybrids – reflected contribution of the 'unknown Western 2' lineage. The non-hybrid accession had a unique substitution (A, consensus G) reflected by a polymorphism (r) in the hybrid accession. Thus, the hybrid most likely was a cross between 'true' *H. pilosum* and an unknown or extinct ('unknown Western 2') species. Both accessions had the same cpDNA which was shared with other members of the '*H. porrifolium*' subclade.

***H. villosum* Jacq.**
In morphology and distribution, it is very similar to *H. pilosum*, but has a less dense involucrum with distinctly broader outer bracts. The species is morphologically variable. A tetraploid accession from Slovakia had few polymorphic sites most of which were shared with other species of the '*H. porrifolium*' subclade to which also its cpDNA corresponded. It could be an autotetraploid derived from extinct diploid or triploid ancestors. A triploid accession from France showed all additive sites typical for interclade hybrids with the 'Eastern' sequence variant strongly dominating. Correspondingly, its DNA content matched that of 'Eastern' species which may indicate backcrossing or unequal proportions of genomes obtained from the parental accessions. Other polymorphisms and cpDNA were shared with species from the '*H. porrifolium*' subclade. This triploid accession had hybrid origin involving a parent from that subclade and an 'unknown Western 2' taxon as pollen donor (see also *H. pilosum*). The species had multiple origin which might be suspected already from its morphological variability.

***H. bupleuroides* C.C. Gmel.**
Like the previous two taxa, this is also a Central European mountain species. Morphologically, it is most similar to *H. porrifolium*. Out of two triploid accessions from Slovakia, one (1212) had few polymorphisms all of which were shared with species of the '*H. porrifolium*' subclade, two of them with *H. porrifolium* itself. Either the species was derived from that diploid taxon or they share a common ancestor. Its cpDNA corresponded to other species of the '*H. porrifolium*' subclade. The second accession (1033) somewhat resembling *H. umbellatum* morphologically was actually introgressed by this species according to seven additive polymorphisms and cpDNA. It had also a higher DNA content (accessions of the '*H. umbellatum*'clade had the highest values among 'Eastern' taxa) than the 'pure' accession (1212) with which it shared five prominent polymorphisms. Additionally, two adjacent polymorphic sites were triple peaks that were additive between polymorphic sites of 1212 and different character states specific for all '*H. umbellatum*' clade taxa (A/T plus G, and T/G plus A). Thus, this accession of *H. bupleuroides* was introgressed by *H. umbellatum*, the only widespread diploid species, which acted as the maternal parent. With respect to these patterns and to its morphology, accession 1033 was most likely misidentified and probably corresponds to the intermediate species *H. virgicaule* Nägeli & Peter, which is supposed to have the inferred parentage.

***H. porrifolium* L.**
This diploid taxon is morphologically rather uniform and occurs predominantly in the Southeastern Alps which are considered as a glacial refuge area. Morphologically, it mostly resembles *H. bupleuroides*. The number of polymorphic sites was small. Most were homoplasious, but two were shared with both accessions of *H. bupleuroides*. The species clustered with 'pure' accessions of the previous three taxa ('*H. porrifolium*' clade) with which it shared morphological, distributional and ecological features, and a particular cpDNA haplotype.

***H. umbellatum* L.**
This is the *Hieracium* species with the largest distribution (Eurasia and North America). It is a tall-growing perennial species, and is predominantly diploid (triploid chromosome counts occur, but are much less frequent). Two diploid accessions had few polymorphic sites most of which were unique, one was shared among the two accessions, with *H. eriophorum*, *H. canadense*, and some hybrids involving the '*H. umbellatum*' clade. Accession um.AM.1 shared two further polymorphisms with *H. eriophorum* and *H. pojoritense*. Together with the following three species, *H. umbellatum* belonged to a well-supported subclade of eastern origin ('*H. umbellatum*' clade) which was also characterized by a particular chloroplast haplotype.

***H. eriophorum* St.-Amans**
This diploid endangered species occurs only along the Atlantic coast in Southwest France. Two accessions shared three prominent species-specific polymorphisms and additionally the above-mentioned ones with *H. umbellatum* to which also the cpDNA corresponded. The species is probably a young offspring of *H. umbellatum*. The particular morphology of *H. eriophorum* could be interpreted as a local adaptation to sand dunes along the sea coast.

***H. canadense* Michx.**

This species of subgenus *Hieracium* exceptionally occurs in North America. Some authors consider it as a subspecies of *H. umbellatum*, a view that does not contradict our data. The triploid accession shared one polymorphic site and its cpDNA haplotype with *H. umbellatum* (and *H. eriophorum*).

***H. virosum* Pall.**

This is a polyploid tall-growing perennial with Eastern European and Siberian distribution and some morphological similarities to *H. sabaudum*. Two triploid accessions had five polymorphic sites of which they shared only one. At that site, the '*H. umbellatum*' clade differed from all non-hybrid taxa which indicates that either *H. virosum* was introgressed by another 'Eastern' species (but no other evidence was found to support this possibility) or that it – or more likely its putative diploid predecessor – could be the oldest species of the subclade in which the polymorphism arose of which the derived character state became fixed in the whole subclade. The Siberian accession (1238) had '*H. umbellatum*' type cpDNA while the more Western Russian accession (vir.1) had a unique variant differing by two mutations.

***H. alpinum* L.**

This is a locally widespread mountain species with unique morphology of which diploid populations occur nowadays only in the Eastern and Southern Carpathians. Two diploid accessions had prominent, but few and mostly unique polymorphisms. The species formed a lineage of its own within the 'Eastern' clade. The chloroplast DNA was also unique and shared only with hybrid taxa with a captured 'original' *H. alpinum* chloroplast.

***H. pojoritense* Woł.**

This is a well circumscribed diploid taxon growing in calcareous crevices in the Eastern Carpathians. Morphology suggests some influence of *H. umbellatum*. Zahn [3] considered it as an 'intermediate' species and placed it between *H. sparsum* and *H. racemosum*. The analyzed accession had a very high number of polymorphic sites, many of which were unique, few were shared with species of the '*H. umbellatum*' clade and some other taxa, but seven reflected synapomorphic substitutions of that clade, and four mirrored *H. alpinum*-specific synapomorphies. *Hieracium umbellatum* (or one of its hybrids, for example *H. racemosum*) is the most likely paternal parent of this hybrid. The *H. alpinum ETS* ribotype was dominating across all positions (about 80% of the total signal), and the chloroplast DNA also corresponded to that species. However, *H. pojoritense* does not show any morphological influence of *H. alpinum*. Also, experimental hybrids between *H. alpinum* and *H. umbellatum* have a different phenotype (P. Mráz, pers. comm.). Taken together with the diploid condition of *H. pojoritense*, the relatively large number of unique polymorphic sites and the species' distribution in a well-known glacial refuge area, it might be concluded that it is an old taxon that either for some reason does not resemble *H. alpinum* morphologically, or that it has originated from an extinct species closely related to, but morphologically different from recent *H. alpinum*. In this context, the captured '*H. alpinum*' cpDNA of *H. sparsum* could be a hint that Zahn's opinion was quite accurate.

***H. petrovae* Vladimirov & Szeląg**

This is a rare, recently described diploid species of section *Pannosa* from the Balkans. Expectedly, it fell into the 'Eastern' clade. It had few polymorphisms; the two most prominent ones were shared only with polyploid *H. pannosum*. Its chloroplast haplotype was the same as that of *H. kittanae* and *H. pannosum*, other Balkan species.

***H. pannosum* Boiss.**

This is a widespread polyploid taxon occurring from the Balkans to Anatolia. It belonged to the 'Eastern' clade and had a rather high number of polymorphisms. Nine out of twelve shared polymorphisms occurred also in other Balkan species, two of these were exclusively shared with *H. petrovae*. An additional polymorphism reflected an autapomorphic substitution of *H. petrovae* with which it also shared its cpDNA haplotype. Thus, the taxon could be a polyploid derivative of *H. petrovae*.

***H. kittanae* Vladimirov**

This is another recently described diploid species. Its has unique morphology, is restricted to a few localities in Bulgaria, and has relict character. Nowadays, few *Hieracia* co-occur with this taxon, but in former times there may have been more species in this area. It formed a separate lineage within the 'Eastern' clade and had the highest number of polymorphic sites of any non-hybrid species. Most were shared with other species from the Balkans. They had apparently accumulated on various different rDNA copies according to cloned sequences (Additional file 2). Its cpDNA corresponded to that of *H. pannosum* and *H. petrovae*.

***H. naegelianum* Pančić**

The species occurs in the Balkan Peninsula and in the Abruzzy Mountains in Central Italy, mostly in refugial areas. It has a rather unique morphology and forms elongated, stolon-like underground rhizoms, an unusual trait in *Hieracium* s.str. The analyzed triploid accession fell into the 'Eastern' clade and had a rather high number of polymorphic sites, many of them unique. Half of the shared polymorphisms were inconclusive as they matched different (unrelated) taxa and hybrids; the other half were shared with a few species and hybrids from the Balkans. Its chloroplast haplotype was also unique. Its morphology, *ETS* polymorphisms, and cpDNA support a rather isolated position within the 'Eastern' clade. While no evidence of introgression from a 'Western' species is apparent from the molecular data, its occurrence in Italian glacial refuges could be indicative of past contacts and introgression from which only an unusually small genome size is left.

***H. sparsum* Friv.**

Hieracium sparsum s.l. occurs in Southeastern Europe, Northern Anatolia and the Caucasus, and comprises morphologically rather different growth forms. In contrast, *H. sparsum* s.str. is diploid, morphologically quite uniform, and is missing in the Caucasus. Two analyzed diploid accessions fell into the 'Eastern' clade and shared two otherwise unique polymorphisms. Apart from these, the *ETS* sequences lacked aut- or syn-apomorphic character states which might indicate that it is a rather old taxon which is also reflected by its position at the base of the 'Eastern' polytomy in the phylogenetic tree. Eight polymorphic sites were shared with other species and hybrids from the Balkans, one with a Romanian accession of *H. alpinum* (alp.Boa.2). Surprisingly, its chloroplast DNA was apparently derived from an '*H. alpinum*' haplotype. The diploid's recent distribution area does not overlap with that of *H. alpinum*. However, polyploid *H. sparsum* (s.l.) is subalpine and known to form hybrid taxa with *H. alpinum*. As morphology of the analyzed accessions does not show any evidence for an introgression of *H. alpinum*, we assume either a chloroplast capture event that has happened very early in the history of the species or an origin from an unknown or extinct species with *H. alpinum* cpDNA, but different morphology (maybe analogous to the situation in *H. pojoritense*).

***H. amplexicaule* L.**

This mountain species is rather widespread in the Alps, in South and Southwest Europe, and North Africa (with numerous secondary occurrences outside its native range). It is morphologically variable with characters connecting it to Pyrenean taxa, but with a much wider ecological amplitude. It forms many intermediates with other taxa. The analyzed triploid accession from Austria showed additivity at positions differing between 'Eastern' and 'Western' clade species. The 'Western' variant was predominant and constituted about 60–70% of the total signal. In addition, it shared six polymorphic sites with Pyrenean taxa indicating that it is a product of a hybridization between a taxon of the 'Pyrenean' lineage and an 'Eastern' clade species. Several polymorphisms were shared with different 'Eastern' species so that its exact origin could not be inferred. Its chloroplast haplotype was derived from one of the 'Pyrenean' variants.

***H. caesium* (Fr.) Fr.**

This is a locally rather widespread, predominantly calciphilous mountain species with mainly North and Central European distribution. The analyzed tetraploid accession from Sweden (microspecies *H. plumbeum* Blytt et Fr.) had hybrid origin and involved a member of the '*H. umbellatum*' group and a 'Western' clade species. The 'Western' ribotype was dominating (about 80–90% relative contribution according to direct sequencing; only one out of six clones represented the '*H. umbellatum*' variant). One polymorphim was exclusively shared with *H. umbellatum* um.AM.1 which is a common genotype according to its inferred

contribution to species and hybrids across a large geographic area. A rare 1 bp-deletion was shared with *H. villosum* 1029. As the only clone showing this feature belonged to the 'Western' ribotype while *H. villosum* was an 'Eastern' clade taxon, this was probably a parallel mutation. The 'Western' parent could not be resolved: only one polymorphic position was shared with many 'Western' species. Morphologically, *H. caesium* resembles *H. bifidum* which might have been involved in its origin. It had a chloroplast of 'Western' origin. Its low 1Cx genome size is indicative of larger amounts of a 'Western' genome.

***H. mixtum* Froel.**

This Pyrenean-Cantabrian mountainous-alpine species is occasionally used as an ornamental plant and has a neophytic occurrence in Germany from which we analyzed one accession. According to published chromosome counts, this species is triploid. Its *ETS* had a relatively high number of small polymorphisms. Alternative character states showed shared polymorphisms for interclade hybrids at all 15 positions. However, neither 'Eastern' nor 'Western' clade-specific character states were dominating, but a mixture of these. This indicates that subsequent to hybridization, the *ETS* arrays became to a large degree homogenized towards a novel unique hybrid sequence. No particular parental taxa could be identified. Its cpDNA was also unique.

***H. prenanthoides* Vill.**

Diploid *H. prenanthoides* is a morphologically rather uniform mountaneous taxon of the Southwestern Alps. As diploids are only known from this area, they can be considered as relict populations that may have survived in this glacial refuge area. Their distribution coincides with a western origin of this species as suggested by the *ETS* data. Polyploids of this species are morphologically variable and occur in subalpine habitats throughout the European montains, the Caucasus and neighboring areas, Central Asia and Siberia; in Northeastern Europe and Siberia also in the lowlands. Their distribution area is large and fragmented. Polyploids form many intermediate types with other species. Zahn [3] supposed morphological influences of section *Cerinthoidea* (mostly Pyrenean taxa) and also similarities to *H. umbellatum*. The three accessions analyzed were similar in sharing two unique polymorphic sites and a strong interclade hybrid signature at the 3'-end of the *ETS* region, and in containing predominantly the 'Western' ribotype. Their cpDNA also belonged to the 'Western' variant or was derived from it. The dominating 'Western' *ETS* ribotype and the geographic distribution of the diploid could hint at an early introgression by a diploid species of eastern origin (or a hybrid contributing an 'Eastern' genome) occurring in the Southwestern Alps. Backcrossing towards *H. prenanthoides* (because of low 'Western' genome size) and a recombination event may have left a trace of the 'Eastern' introgressant's genome in a predominantly 'Western' *ETS* background. This signature was still present in the triploid accessions that arose later from the diploids of this species by further hybridization: The triploid accession 1161 showed additionally the 'unknown Western 1' *ETS* variant, suggesting introgression by a further, potentially extinct taxon. The triploid 1187 lacked this variant, but instead showed subsequent introgression by an 'Eastern' clade taxon. More specifically, part of the additional polymorphisms were shared with the '*H. umbellatum*' group, and cloned recombinant sequences suggested that the other part was lost from the genome (Additional file 2: Patterns of *ETS* recombination). Thus, *H. prenanthoides* seems to have a cryptic history of reticulation between different clades which in case of the triploids involved at least two subsequent hybridization events. The morphological variability of polyploids of this species may be due to recurrent origin from diploid *H. prenanthoides* hybridizing with different other taxa. Whether the initial 'Eastern' introgressant also belonged to the '*H. umbellatum*' clade cannot be decided, because the 3'-end bearing the 'Eastern' hybrid signature contains no substitutions distinguishing between the '*H. umbellatum*' subclade and other 'Eastern' taxa.

***H. lachenalii* Suter**

The species is widespread across all of Europe and Western Asia, morphologically very polymorphic and mostly lacking in specific characters. It fills the morphological space between *H. murorum* and *H. laevigatum*. According to *ETS* sequence, the triploid accession belonged to the 'Western' clade, but its cpDNA matched that of the '*H. umbellatum*' group to which the species has some morphological similarity. However, not a single polymorphic site specific for that group was present in its *ETS* sequence. This suggests

ancient introgression by *H. umbellatum* (or a sexual hybrid carrying an '*H. umbellatum*' genome) with either almost complete homogenization of *ETS* towards the 'Western' clade (only two polymorphic positions showed a small amount of general 'Eastern' clade character states) or repeated backcrossing to a 'Western' species prior to polyploidization. Its higher DNA content – about 5% above the usual values for 'Western' clade species – may be a consequence of that introgression. With respect to its morphological variability and poor taxonomic circumscription, it is likely that *H. lachenalii* is composed of types with multiple origin from similar species combinations.

***H. laevigatum* Willd.**

Like *H. lachenalii*, this is a widespread and highly polymorphic species, but with prominent morphological similarity to *H. umbellatum*. All 15 sites differing between clades were additive in the analyzed triploid accession. In addition, it shared seven polymorphisms or substitutions with the '*H. umbellatum*' group. Its chloroplast DNA also belonged to that group. Thus, the accession had hybrid origin involving a genetically undifferentiated 'Western' clade species and a member of the '*H. umbellatum*' group.

***H. racemosum* Waldst. et Kit. ex Willd.**

This is a very polymorphic species, some accessions resembling *H. lucidum*, some *H. sabaudum*. With the latter species, it forms a morphological continuum, but has a more southern distribution (East-Submediterranean to Southern Silesia, further east in northern areas, overlapping with *H. virosum*). The analyzed triploid accession showed additive patterns between 'Eastern' and 'Western' clade taxa with the 'Eastern' variant slightly dominating. In addition, eight character states or polymorphisms as well as its chloroplast haplotype were shared with the '*H. umbellatum*' group. There was also a strong contribution of the 'unknown Western 1' sequence variant at all four positions diagnostic for that type. Thus, this unsampled or extinct variant reflected the 'Western' clade parent, and a species of the '*H. umbellatum*' group or a hybrid involving that group provided the 'Eastern' *ETS* ribotype. Its morphological similarity with *H. lucidum* or *H. sabaudum* could be due to the shared 'unknown Western 1' genome; its high variability might be caused by multiple origins involving different additional taxa.

***H. sabaudum* L.**

It has a wider geographic distribution than *H. racemosum* in northern, eastern and western directions. The involucral bracts of both species are similar. Also, *ETS* character additivity between both major clades, contribution of the 'unknown Western 1' sequence variant, and sharing of character states, polymorphisms and cpDNA with the '*H. umbellatum*' group of the triploid accession analyzed corresponded to *H. racemosum*. Additional polymorphic sites narrowed the parentage of the '*H. umbellatum*' group down to *H. umbellatum* um.AM.1 sampled from the same locality. In accessions of both *H. sabaudum* and *H. racemosum*, the 'Eastern' sequence variant slightly predominated according to relative peak heights, more strongly so in *H. sabaudum*. Correspondingly, two out of three clones in *H. sabaudum* and three out of six clones in *H. racemosum* reflected the '*H. umbellatum*' ribotype.

***H. bracteolatum* Sibth. & Sm.**

This species has a unique morphology, belongs to a monotypic section, and occurs only in Greece. Nevertheless, the analyzed triploid accession had the same inferred parentage as the previous two taxa (but lacking the additional polymorphisms of *H. sabaudum*). 'Western' and 'Eastern' sequence variants were present in about equal amounts. Either the morphological differences despite similar parentage reflect ecological adaptations that did not affect nrDNA variation, or the interspecific genetic variation was too low to distinguish between morphologically different taxa of the same subclade (as in the case of *H. umbellatum* and *H. eriophorum*).

***H. glaucum* All.**

Morphologically, this species shows influences of *H. bifidum* and either *H. porrifolium* or *H. bupleuroides*. The analyzed triploid accession was indeed an interclade hybrid. Two additional additive sites showed contribution of the '*H. porrifolium*' subclade. Another polymorphism was shared with *H. porrifolium* and *H. bupleuroides*. In addition, a three-character state additive site composed of a further polymorphism shared by

these two species and a ‘Western’ clade substitution occurred (W + C). CpDNA also corresponded to the ‘*H. porrifolium*’ haplotype. The ‘Western’ clade was less differentiated, and there were only few sites available for tracing the second parent. However, at a single site, *H. glaucum* shared a polymorphism with *H. bifidum* and *H. humile* 1064. Thus, in this case, the *ETS* data reflected the morphological similarities.

***H. olympicum* Boiss.**

This is a species from the Southern Balkans and Northern Anatolia. The triploid accession showed character additivity at all 15 positions distinguishing ‘Eastern’ and ‘Western’ ribotypes. The ‘Eastern’ variant was somewhat dominating, especially towards the 3’-end, which was reflected by the recombinant sequences (Additional file 2: Patterns of *ETS* recombination). The ‘Western’ clade was represented by the ‘unknown Western 1’ ribotype. Eight additional polymorphisms were shared with different species from the Balkans. The most similar pattern belonged to *H. sparsum*. This combination of ‘Eastern’ and ‘Western’ subtypes was unique among all analyzed accessions. Its cpDNA was also unique, most similar to that of *H. gymnocephalum*, another interclade hybrid. However, none of the identified ribotypes were shared among these two taxa suggesting the involvement of an unknown maternal parent not reflected by *ETS* for at least one of them.

***H. gymnocephalum* Griseb. ex Pant.**

The species occurs in the Western Balkans. In Macedonia, its distribution area overlaps with *H. olympicum*. The analyzed accessions – one of them diploid, one triploid – indicated this to be also a hybrid taxon between the ‘Eastern’ and ‘Western’ clade. Apart from the interclade additive sites, a rather large number of additional intra-individual polymorphisms occurred. Eight (4 + 4) represented the ‘unknown Western 2’ and the ‘unknown Eastern’ ribotypes (Additional file 2). Only 1–2 polymorphisms were shared with ‘pure’ species from the Balkans (Figure 4). A unique cpDNA haplotype and a unique substitution in the *ETS* (note that substitutions were generally rare and most of the variation within clades was based on shared polymorphisms) suggested that this might be a rather old taxon and, in addition, that perhaps no extant species can be considered as a direct predecessor. It is also remarkable that despite different ploidy levels, the two accessions had a nearly identical pattern of intra-individual polymorphisms and equal ratios of ‘Eastern’ and ‘Western’ ribotypes. Nearly identical, but rather derived cpDNA haplotypes of *H. olympicum* and *H. gymnocephalum* indicate a common maternal origin of these species from an unknown donor. It may have been a taxon belonging to the ‘unknown Eastern’ lineage according to geographic distribution. In any case, either *H. olympicum* or *H. gymnocephalum* (or both) must have had an additional genome donor that could not be traced as their parentages inferred from *ETS* ribotypes showed no overlap while their cpDNAs matched.

***H. heterogynum* (Froel.) Guterm.**

This species has a similar distribution to *H. gymnocephalum*, mostly occurring in former Yugoslavia. Morphologically, it is characterized by a particular kind of indumentum. The analyzed accession was also an interclade hybrid. A high number of additional shared polymorphisms occurred in the *ETS*. Many were accession-specific or occurred at homoplasious positions. Four reflected the ‘unknown Eastern’ ribotype and were shared with the interclade hybrids *H. gymnocephalum* and *H. plumulosum*. Two polymorphisms showed additivity with species of the ‘*H. umbellatum*’ group to which also the cpDNA corresponded. Apparently, an ‘Eastern’ species from the ‘*H. umbellatum*’ group was involved in its origin, but only two out of seven sites in the *ETS* (plus the cpDNA) reflected this. Both remaining character states of the ‘*H. umbellatum*’ ribotype were situated on the ‘wrong’ strands suggesting that their character states were probably maintained by gene conversion while the other five were lost from the genome (Additional file 2), maybe together with a particular rDNA locus. An ‘*H. umbellatum*’ chloroplast haplotype in combination with an almost erased ‘*H. umbellatum*’ *ETS* pattern also suggests that this clade made a rather ancient genome contribution to *H. heterogynum* and that several subsequent hybridization events occurred through pollination by different donors. A clone representing the ‘unknown Western 2’ ribotype was also found (in addition to ‘ordinary’ ‘Western’ sequences) although exceptionally direct sequencing did not show any trace of this, i.e., this ribotype must be present in less than 5% of all copies in the genome. Further accession-specific polymorphisms were retrieved by cloned sequences that were not present in the direct sequence, but the clones

also did not retrieve all of the accession-specific substitutions inferred from direct sequencing. It seems that this accession has particularly complex rDNA arrays and, in addition to the two '*H. umbellatum*'-specific characters, more examples of potential gene conversion were found (Additional file 2). In the triploid accession of *H. heterogynum*, three different ribotypes could be clearly identified, plus remnants of a fourth whose genomic contribution was also apparent from cpDNA.

***H. plumulosum* A. Kern.**

This species occurs in the Western Balkans. Species with a similar kind of indumentum are known from the Southwestern Alps and from the Appennin. But as this is a rather widespread feature of *Hieracia* in Submediterranean regions, it could be an adaptation to dry climate instead of reflecting species relationships. Morphologically, *H. plumulosum* stands between *H. pannosum*-like species and possibly *H. tomentosum*. It also had a genome composed of the two major clades, however, the 'Eastern' ribotype was strongly overrepresented (about 80% of the total signal). The analyzed diploid from a deep river valley with relict character had by far the highest number of polymorphic sites among all investigated accessions (37). Many of them were unique, but it also shared more polymorphic sites with other taxa than most samples. In addition to 'ordinary' 'Western' and 'Eastern' variants, the 'unknown Western 2' and the 'unknown Eastern' ribotypes were found (Additional file 2). The latter was shared with the interclade hybrids *H. heterogynum* and *H. gymnocephalum* and may indicate a genome donor geographically restricted to the Balkans. This is supported by 1–2 'Balkan' polymorphisms occurring on the 'unknown Eastern' strands suggestive of the origin of this ribotype from a Balkan species. Its chloroplast haplotype was unique and comparably divergent from all other species. This taxon (or at least this accession) had a highly reticulate history with four *ETS* ribotypes and a chloroplast haplotype not corresponding to any other taxon sampled. This example points out that at least two major rDNA loci per haploid genome should exist.

Details of accession origin (including outgroup taxa)

Species	Accession	Origin of samples
H. alpinum L.	alp.Ukr	Ukraine: Chornohora Mts., Ukrainian Carpathians, Polonina Breskulska ridge, saddle between Mt. Hoverla and Mt. Breskul, 1800 m a.s.l., 48°09'09.8''N, 24°30'14.6'', 23 July 2003, leg. P. Mráz & J. Chrtek
	alp.Boa.2	Romania: Munții Rodnei Mts, glacial cirque on the NE slopes of Mt. Pietrosul Mare, ca 0.3 km SE from Stația Meteo, ca 1900 m a.s.l., 5 July 2001, leg. P. Mráz
H. amplexicaule L.	1050	Austria: Carinthia, Hohe Tauern, Goldberggruppe: Innerfragant, near the old (unmarked) path to the Fraganter Hütte, ca 1 km SW of the village, rocks above the brook, 1233 m, 13º 04´20´´ E, 46º 57´56´´ N, 28 July 2005, leg. J. Chrtek & P. Mráz
H. bifidum Kit.	1213	Slovakia: Orava, distr. Tvrdošín, Oravice: Juráňova dolina-Tiesňavy, 3 km SE of the village, limestone gorge, 930 m, 19º 46´19´´ E, 49º 16´31´´ N, 15 August 2006, leg. J. Chrtek
H. bracteolatum Sibth. & Sm.	1240	Greece: Thessalia, Pilion Mts., Agriolefkes, 1400 m, leg. Binder et al. (S, BGBM Berlin-Dahlem)
H. bupleuroides C.C. Gmel.	1033	Slovakia: distr. Ilava, Biele Karpaty Mts.: Vršatské Podhradie, castle ruine of Vršatec, 770 m, 18º 09´00´´ E, 49º 03´57´´ N, 15 June 2005, leg. J. Chrtek
	1212	Slovakia: Orava, distr. Tvrdošín, Oravice: Juráňova dolina-Tiesňavy, 3 km SE of the village, limestone gorge, 930 m, 19º 46´19´´ E, 49º 16´31´´ N, 15 August 2006, leg. J. Chrtek
H. caesium (Fr.) Fr.	1231	Sweden: prov. Gotland, par. Hall, open limestone scree by the sea 1.3 km SE of Hallshuk (close to the NW point of the island of Gotland ca 40 km NNE of Visby), 20 m, 18º 43´ E, 57º 55´ N, July 2006, leg. et det. T. Tyler & A. Sennikov
H. canadense Michx.	canad	Canada: B&T World Seed, http://www.b-and-t-world-seeds.com/index.html, herbarium GLM 157756
H. candidum Scheele	1197	Spain: Catalunya, prov. Lérida, distr. La Seu d´Urgell: Adraén, Serra del Cadí mountain ridge, NW slopes, 3 km SE of the village, 1750 m, road margin in a pine forest, 23 July 2006, leg. J. Chrtek, G. Mateo & J. A. Rosselló, det. G. Mateo
H. cerinthoides L.	1176	Spain: Catalunya, prov. Lérida, Pirineus Mts: Os de Civís, 1 km WSW of the village, margin of a pasture, 1720 m, 1º 25´46´´ E, 42º 26´47´´ N, 21 July 2006, leg. J. Chrtek, G. Mateo & J. A. Rosselló, det. G. Mateo
H. cordifolium Lapeyr.	1177	Andorra: Pirineus Mts, Bixessarri (NW of Sant Julià de Lòria), valley of Torrent dels Llimois, rocks and margins of a path ca 100 m from the street, 1.5 km NW of the village, 1305 m, 1º 26´44´´ E, 42º 29´33´´ N, 21 July 2006, leg. J. Chrtek, G. Mateo & J. A. Rosselló, det. G. Mateo
H. eriophorum St.-Amans	1221	France: dépt. Landes, Labenne: plage de Labenne Océan Sud, 10 m, 1º 27´20´´ W, 43º 36´17´´ N, 27 September 2006, leg. E. Forey
	1222	France: dépt. Landes, Seignosse-le-Penon, plage de Estagnols Seignosse, 10 m, 1º 25´51´´ W, 43º 41´40´´ N, 27 September 2006, leg. E. Forey
H. glaucum All.	1230	Slovenia: Julian Alps, village of Trenta: Zadnja Trenta valley, 0.5 km W of the chalet 'Koča pro izviru Soče', margin of gravel alluvium, 910 m, 13º 43´41´´ E, 46º 24´22´´ N, September 2006, leg. J. Chrtek
H. gouani Arv.-Touv.	1171	Spain: Catalunya, prov. Gerona: rocks at the road between Ripoll and Ribes de Fresser, 1290 m, 2º 10´02´´ E, 42º 15´27´´ N, 24 July 2006, leg. J. Chrtek
H. gymnocephalum Griseb. ex Pant.	1215	Albania: NW part, Jezercës, 21 km NW of Bajram Curri, 1800 m, 19º 50´23´´ E, 42º 25´46´´ N, August 2006, leg. J. Zahradníček, det. Z. Szeląg & V. Vladimirov
	1207	Montenegro: Durmitor Mts, August 2006, leg. and det. Z. Szeląg
H. gymnocerinthe Arv.-Touv. & G. Gaut.	1172	Spain: Catalunya, prov. Lérida, distr. La Seu d'Urgell: Adraén, Serra del Cadí mountain ridge, NW slopes, 1 km SE of the village, 1600 m, road margin in a pine forest with dominating *Arctostaphylos uva-ursi*, 1º 30´34´´ E, 42º 16´15´´ N, 23 July 2006, leg. J. Chrtek, G. Mateo & J. A. Rosselló, det. G. Mateo
H. heterogynum (Froel.) Guterm.	1250	Montenegro: Kotor, Mt. Lovčen, 1350 m, 18º 47´ E, 42º 23´ N, 20 August 2006, leg. M. Niketić
H. humile Jacq.	1064	Austria: Oberösterreich, Dachstein massif: Vorderer Gosausee (mountain lake), rocks on NW bank, 6 km SSW of the village of Gosau, 940 m, 13º 29´55´´ E, 47º 31´53´´ N, 13 August 2005, leg. J. Chrtek
	1188	France: Le Midi, dépt. Aude, Corbières Mts: Bugarach, Mt. Pech de Bugarach, 1130 m, 2º 22´47´´ E, 42º 52´06´´ N, 27 July 2006, leg. J. Chrtek
H. kittanae Vladimirov	1228	Bulgaria: Central Rhodope Mts.: Trigrad gorge, limestone rocks near the natural entrance to Dyavolskoto garlo cave, 750–800 m, September 2005, leg. P. Ignatova
H. lachenalii Suter	1160	Czech Republic: Moravia, distr. Znojmo: Lukov, forest 1.3 km SSW of the village, 410 m, 15º 54´27´´ E, 48º 51´04´´ N, June 2006, leg. J. Zahradníček
H. laevigatum Willd.	1031	Czech Republic: Bohemia, distr. Rokycany: Strašice, N part of the village, margin of a forest, 550 m, 13º 45´07´´ E, 49º 44´51´´ N, 29 June 2005, leg. J. Chrtek
H. lawsonii Vill.	1175	France: Le Midi, dépt. Aude, Corbières Mts: Bugarach, Mt. Pech de Bugarach, 1130 m, 2º 22´47´´ E, 42º 52´06´´ N, 27 July 2006, leg. J. Chrtek
H. lucidum Guss.	H.lucidum	Italy: Sicily, distr. Palermo: Sferracavalio, limestone rocks between the village and Capo Gallo, 40 m, 13º 17´56´´ E, 38º 12´56´´ N, 11 April 2007, leg. J. Chrtek et al.
H. mixtum Froel.	H.mixtum	Germany: Lower Saxony, 3723/33, southern part of the Deister hill near Springe, quarry at the Ebersberg, 345 m, herbarium GLM
H. murorum L.	875	Czech Republic: Bohemia, distr. Plzeň: Plzeň, village of Koterov, street "V závrtku", 0.6 km SSW of the railway station "Plzeň-Koterov", slopes along the street, ca 320 m, 13º 25´07´´ E, 49º 43´02´´ N, 12 August 2003, leg. M. Král
H. naegelianum Pančić	1208	Montenegro: Durmitor Mts.: Mt. Veliki Međed, alpine grassland on limestone, 2050 m, 43º 03´31´´ N, 19º 04´13´´ E, 1 August 2006, leg. and det. Z. Szeląg
H. olympicum Boiss.	1206	Bulgaria: Stara Planina Mts., Kaloferska Planina Mts.: Valley of Vidima riiver, 2 km NE of the Kaloferski Monastyr, eroded slope in *Carpinus orientalis* forest, 870 m, 24º 58´42´´ E, 42º 40´36´´ N, 9 August 2006, leg. and det. Z. Szeląg
H. pannosum Boiss.	1205	Bulgaria: Stara Planina Mts., Trojanska Planina Mts.: Mt. Kozja stena, grassy slope on limestone, 1570 m,

		42°47´27´´ N, 24°34´06´´ E, 8 August 2006, leg. and det. Z. Szeląg
H. petrovae Vladimirov & Szeląg	1229	Bulgaria: Central Rhodope Mts.: Trigrad gorge, crevices of limestone rock (*locus classicus*), 750–800 m, 41°39′ 55´´N, 24°21′ 50´´E, 15 October 2005, leg. V. Vladimirov
H. pictum Pers.	1067	France: dépt. Alpes Maritimes, valley of la Roya: Tende, along the old road to the Col de Tende, ca 0.5 km above the tunnel, 6 km NNW of the village, 1331 m, 07º 33´ 57´´ E, 44º 08´ 19´´ N, 28 August 2005, leg. J. Chrtek & P. Mráz
	1307	France: dépt. Hautes Alpes, Briançon, near a path to Fort des Sallettes, 1360 m, 6º 38´ 52´´ E, 44º 54´ 33´´ N, 2 August 2007, leg. J. Chrtek et al.
H. pilosum Schleich. ex Froel.	1226 (2)	Slovenia: Julijske Alpe Mts, Log pod Mangartom: Mt. Travnik (2200 m), SW slopes, limestone, 2120 m, 13º 38´40´´ E, 46º 26´22´´ N, 7 September 2006, leg. J. Chrtek, J. Fehrer et al.
H. plumulosum A. Kern.	1218	Montenegro: Canyon of the Mrtvica river, 35 km SW of Kolasin, halfway through the canyon, near the bridge, 1000 m, 19° 48´ 59´´ E, 42° 28´ 40´´ N, August 2006, leg J. Zahradníček, det Z. Szeląg
H. pojoritense Woł	poi.Rom	Romania: Pojorita, Cimpalung Moldovec, herbarium P. Mráz
H. porrifolium L.	1052	Austria: Carinthia, the Karawanken Mts.: Bad Eisenkappel, limestone rocks and pine forests (alliance *Erico-Pinion*) near the road to Bad Vellach, 4.5 km SSW of the town, 658 m, 14º 34´20.5´´ E, 46º 27´07.1´´ N, 26 July 2005, leg. J. Chrtek & P. Mráz
H. prenanthoides Vill.	1161	Poland: Województwo dolnośląskie, Karkonosze Mts., Jagniątków: Mały Kocioł Śnieżny glacial cirque, along the path, 1250 m, 15° 33´ 28´´ E, 50° 46´ 27´´ N, July 2006, leg. J. Zahradníček
	1252	France: dépt. Hautes Alpes, La Grave, below the village, ca 1500 m, 06º 18´21´´ E, 45º 02´37´´ N, June 2003, leg. P. Mráz
	1187	Andorra: Pirineus Mts, Canillo, SE margin of the village, 1530 m, 1º 36´11´´ E, 42º 33´56´´ N, 22 July 2006, leg. J. Chrtek, G. Mateo & J. A. Rosselló
H. racemosum Waldst. et Kit. ex Willd.	874	Czech Republic: SW Moravia, distr. Znojmo, Vranov nad Dyjí, forest 0.6 km SE of the village, 370 m, 15º 49´09´´ E, 48º 53´32´´ N, 27 September 2003, leg. J. Chrtek
H. ramondii Griseb.	1173	Andorra: Pirineus Mts., Encamp, valley of Riu de les Deveses, NW slopes of Mt. Alt del Griu, 3.8 km E of the town, rocky outcrops in a light mountain forest, 2040 m alt., 1º 37´52.1´´ E, 42º 32´07.1´´ N, 22 July 2006, leg. J. Chrtek, G. Mateo & J. A. Rosselló, det. G. Mateo
H. recoderi De Retz	1174	Spain: Catalunya, prov. Barcelona: Berga, monastery of Queralt, rocks ca 200 m below the parking place, 805 m, 1º 49´24´´ E, 42º 06´54´´ N, 24 July 2006, leg. J. Chrtek
H. sabaudum L.	1098	Germany: Oberlausitz , distr. Löbau-Zittau: Schönau-Berzdorf, 220 m, 14º 53´48´´ E, 51º 04´01´´ N, July 2004, leg. S. Bräutigam
H. schmidtii Tausch	1025	Czech Republic: Bohemia, distr. Litoměřice: Boreč, the Boreč hill, E slope, 500 m N of the village, 367 m, 13º 59´25´´ E, 50º 30´53.4´´ N, 15 May 2005, leg. J. Chrtek
H. sparsum Friv.	1251	Bulgaria: Sofia, Vitoša Mts.: NE slope of Mt. Vitoša, Bistriško Branište biosphere reserve, 2000 m, 23º 17´56´´ E, 42º 34´07´´ N, 23 June 2006, leg. F. Krahulec & A. Krahulcová
	spa.sst.2	Bulgaria: Pirin Mts., Vihren Mt., garden culture Z. Szeląg, herbarium PRA
H. stelligerum Froel.	1233	France: dépt. Ardèche, Vallon Pont d`Arc: crevices of calcareous rocks along the road D 390, opposite of ‘le Pont d'Arc’, ca 3.5 km SE of the village, 500 m, 04º 24´ 10´´ E, 44º 24´ 25´´ N, October 2006, leg. P. Mráz
H. tomentosum L.	1066	France: dépt. Alpes Maritimes, valley of la Roya: Tende, along the old road to the Col de Tende, ca 0.5 km above the tunnel, 6 km NNW of the village, 1331 m, 07º 33´ 57´´ E, 44º 08´ 19´´ N, 28 August 2005, leg. J. Chrtek & P. Mráz
H. transylvanicum Heuff.	1077	Ukraine: Oblast´ Zakarpatska, Marmaros´ki Al´py Mts.: Mt. Berlebashka (1480 m), NW slope along the trail (red marked), E of the village of Dilove, 1200 m, 24º 21´31´´ E, 47º 56´13´´ N, 19 September 2005, leg. J. Zahradníček
	tra.Boa	Romania: Munții Rodnei Mts, border of the tourist path from the village of Borşa to Mt. Pietrosul Mare, spruce forest, 1300–1400 m a.s.l., 47°39’ N, 24°39’ E, 5 July 2001, leg. P. Mráz
H. umbellatum L.	1021	Poland: Województwo pomorskie, Baltic coast, Jantar, 5 m, 19º 02´27´´ E, 54º 20´00´´ N, 27 June 2002, leg. et det. Z. Szeląg
	um.AM.1	Germany: Upper Lusatia, SE Schönau-Berzdorf, herbarium GLM 46889
H. villosum Jacq.	1029	Slovakia: Strážovské vrchy Mts, distr. Ilava: Mt. Strážov, summit region, calcareous rocks, 1310 m, 18º 27´44´´ E, 48º 57´19´´ N, 18 June 2005, leg. J. Chrtek
	1305	France: dépt. Savoie, slopes below (N of) Col du Galibier, the mountain lake 10 km S of the village of Valloire, 2380 m, 6º 24´43´´ E, 45º 04´21´´ N, 3 August 2007, leg. J. Chrtek et al.
H. virosum Pall.	vir.1	Russia: Rostov-na-Donu, vicinity of the town, 150 m, 39º 49´41´´ E, 47º 17´05´´ (Botanical Garden Rostov)
	1238	Russia: Siberia, Altajskij kraj, W of Barnaul, 220 m, leg. Ristow/Seitz (BGBM Berlin-Dahlem)
Pilosella lactucella (Wallr.) P. D. Sell & C. West	lac.Jon.1	Germany: Erzgebirge, culture Görlitz, herbarium GLM 140613
Hispidella hispanica Barnades ex Lam.	His.his.2	Spain: Sierra de Guadarrama, leg. J. Pizarro et C. Navarro no CN 2460, herbarium M
Andryala integrifolia L.	Ant.int.2/2	Spain: Andalusia, Malaga, herbarium GLM 141138
Andryala levitomentosa (Nyár.) P.D. Sell	A.lev.maj.1	Romania: Pietrosul Bogolini, herbarium GLM 156367
‘*Hieracium*’ *intybaceum* All.	H.intybac	B&T World Seed, http://www.b-and-t-world-seeds.com/index.html, herbarium PRA
	inb.Kaer	Austria: Kärnten, S. Jagalski 4, herbarium M
