# Supplementary material for: Intra-individual polymorphism in diploid and apomictic polyploid hawkweeds (Hieracium, Lactuceae, Asteraceae): disentangling phylogenetic signal, reticulation, and noise
Source: BMC Evol Biol. 2009 Sep 22;9:239. doi: 10.1186/1471-2148-9-239 (PMC2759941; doi:10.1186/1471-2148-9-239)
Supplement: Additional file 2 — Patterns of ETS recombination. Cloned sequences of nine interclade hybrid accessions (H. olympicum, H. mixtum, H. prenanthoides 1187, H. caesium, H. pilosum 1226/2, H. villosum 1305, H. gymnocephalum 1215, H. heterogynum, H. plumulosum) and H. kittanae, the non-hybrid accession with the highest number of intra-individual polymorphisms, are shown. Cloned sequences are compared with intra-individual polymorphisms and dominant character states revealed by direct sequencing. All cloned accessions with recombinant sequences are included. Patterns of gene conversion and the distribution of accession-specific polymorphisms across the cloned sequences can be traced. Color coding of diagnostic character states as in Figure 5. [file 1471-2148-9-239-S2.PDF]

Additional file 2: Patterns of *ETS* recombination

All cloned accessions with partly recombinant sequences are shown. Results from direct sequencing ('polymorphic', 'dominant peaks') and some additional accessions with non-recombinant clones are for comparison. Positions correspond to Additional files 3 and 4; approximate recombination points inferred by visual inspection are indicated by bold vertical bars.

H. olympicum	**169**	**180**	**187**	**194**	**196–197**	**215**	**233**	**244**	**252**	**254**	**262**	**276**	**281**	**307**	**358**	**368**	**375**	**385**	**390**	**434**	**446**	**461**	**468**	**478**	**479**	**486**	**488**	**514**
polymorphic	W	R	K	M	TC/– –	R	Y	K	Y	Y	T/–	W	Y	R	K	Y	y	Y	R	R	R	W	R	Y	R	W	W	S
dominant peaks	W	R	T	A	TC	R	Y	K	Y	Y	–	W	Y	A	K	Y	T	C	A	G	A	A	G	C	A	T	A	C
clone 5 – Wx	T	A	G	C	– –	G	C	G	C	T	T	T	T	G	T	C	T	T	G	A	G	T	A	T	G	A	T	G
clone 1 – Wx/EB	T	A	G	C	– –	G	C	G	C	T	T	T	T	G	T	C	T	T	G	A	│A	A	G	C	A	T	A	C
clone 4 – Wx/EB	T	A	G	C	– –	G	C	G	C	T	T	T	T	G	│G	T	T	C	A	G	A	A	G	C	A	T	A	C
clone 6 – EB	A	G	T	A	TC	G	T	T	T	C	–	T	C	A	G	T	C	C	A	G	A	A	G	C	A	T	A	C
clone 3 – EB	A	G	T	A	TC	G	T	T	T	C	–	A	C	A	G	T	T	C	A	G	A	A	G	C	A	T	A	C
clone 8 – EB	A	G	T	A	TC	A	T	T	T	C	–	A	C	A	G	T	T	C	A	A	A	A	G	C	A	T	A	C
clone 7 – EB	A	G	T	A	TC	A	T	T	T	C	–	A	C	A	G	T	T	C	A	G	A	A	G	C	A	T	A	C
clone 2 – EB	A	G	T	A	TC	A	T	T	T	C	–	A	C	A	G	T	T	C	A	G	A	A	G	C	A	T	A	C

Color coding in clones: black: consensus; 'Western': blue, 'unknown Western 1': light green, 'Eastern': red, 'Balkan': orange, accession-specific: turquoise

y at 375 is shared with some 'H. porrifolium' *clade species and one of their hybrids; C is underrepresented and probably homoplasious. R at 479 is shared with the* 'H. umbellatum' *clade and their hybrids; as G occurs on the 'Western' strand, it is probably also a homoplasy. Both are therefore labeled here as accession-specific along with 'truely' unique ones.*

Clones 1 and 4 are different recombinants. The dominant peaks in direct sequencing are better resolved towards the 3' end which reflects this recombination pattern.

H. mixtum	**45**	**162**	**183**	**187**	**194**	**197**	**202**	**233**	**244**	**252**	**262**	**281**	**298**	**307**	**341**	**344**	**368**	**446**	**461**	**478**	**485**	**486**	**488**	**515**	**517**
polymorphic	s	y	k	k	m	y	k	y	k	y	T/–	y	y	r	y	y	y	r	w	y	y	w	w	m	k
dominant peaks	G	C	T	G	C	C	G	C	G	C	–	T	C	A	T	T	T	G	T	C	C	T	A	A	T
clone 1 – mix/W	C	C	T	G	C	C	G	C	G	C	–	T	T	A	T	T	T	G	T	│T	T	A	T	C	G
clone 4 – mix/W	C	C	T	G	C	C	G	C	G	C	–	T	T	A	T	T	T	G	T	│T	T	A	T	C	G
clone 8 – mix	C	C	T	G	C	C	G	C	G	C	–	T	C	A	C	T	T	G	T	C	C	T	A	A	T
clone 5 – mix	G	C	T	G	C	C	G	C	G	C	–	T	C	A	T	T	T	G	T	C	C	T	A	A	T
clone 10 – mix	G	C	T	G	C	C	G	C	G	C	–	T	C	A	T	T	T	G	T	C	C	T	A	A	T
clone 9 – mix	G	C	T	G	C	C	G	C	G	C	–	T	C	A	T	T	T	G	T	C	C	T	A	A	T
clone 12 – mix	G	C	T	G	C	C	G	C	G	C	–	T	C	A	T	T	T	G	T	C	C	T	A	A	T
clone 13 – mix	G	C	T	G	C	C	G	C	G	C	–	T	C	A	T	T	T	G	T	C	C	T	A	A	T
clone 14 – mix	G	C	T	G	C	C	G	C	G	C	–	T	C	A	T	T	T	G	T	C	C	T	A	A	T
clone 2 – mix	G	C	T	G	C	C	G	C	G	C	–	T	C	A	T	C	T	G	T	C	C	T	A	A	T
clone 7 – mix	G	C	G	G	C	C	G	T	G	C	–	T	C	A	T	T	T	G	T	C	C	T	A	A	T
clone 6 – E/mix	G	C	G	T	A	C	G	│C	G	C	–	T	C	A	T	T	T	G	T	C	C	T	A	A	T
clone 11 – E/mix	G	C	G	T	A	C	G	│C	G	C	–	T	C	A	T	T	T	G	T	C	C	T	A	A	T
clone 3 – E	G	T	G	T	A	C	T	T	T	T	–	C	C	A	T	C	T	A	A	C	C	T	A	C	G

Color coding in clones: black: consensus; 'Western': blue, 'Pyrenean': dark violet, 'Eastern': red, accession-specific: turquoise

y at 485 is shared with several 'Western' species, T is 'Pyrenean'-specific. k at 202 is shared with H. gouani, *another interclade hybrid. Shared polymorphisms at 45, 162, 344, and 515 are probably homoplasious (see Additional file 3). For simplicity, they are treated here as accession-specific.*

The majority of clones corresponds to the dominant sequence revealed by direct sequencing; these are also characterized by *H. mixtum*-specific nucleotides at 515 and 517. Other 'accession-specific' characters are scattered among them; clone 7 shows the 'Eastern' character at position 233, probably due to gene conversion. Four clones are recombinant (two identical ones each): clones 1 and 4 show the 'Western' signature at the end, clones 6 and 11 show the 'Eastern' type at the beginning. Clone 3 corresponds entirely to an 'Eastern' variant. A pure 'Western' clone – or a recombinant with only 'Western' character states before position 446 – was not found among the sequenced clones, but polymorphisms in the direct sequence (at 197, 262, 307, and 368) indicate their presence in the genome. All 'accession-specific' polymorphisms are accounted for by these clones.

Additional file 2: Patterns of *ETS* recombination

All cloned accessions with partly recombinant sequences are shown. Results from direct sequencing ('polymorphic', 'dominant peaks') and some additional accessions with non-recombinant clones are for comparison. Positions correspond to Additional files 3 and 4; approximate recombination points inferred by visual inspection are indicated by bold vertical bars.

***H. prenanthoides* 1187**	**44**	**187**	**190**	**194**	**195**	**197**	**205**	**232**	**233**	**244**	**252**	**253**	**262**	**281**	**307**	**326**	**349**	**368**	**435**	**446**	**448**	**461**	**478**	**485**	**486**	**488**	**496–505**
polymorphic	y	k	r	m	y	y	y	k	y	k	y	s	T/–	y	r	w	k	y	y	r	y	w	Y	y	W	W	10 bp/—
dominant peaks	T	G	A	C	T	T	T	G	C	G	C	G	T	T	G	T	G	C	T	G	T	T	Y	C	W	W	10 bp
clone 2 – W	T	G	A	C	T	T	T	G	C	G	C	G	T	T	G	T	G	C	T	G	T	T	T	C	A	T	10 bp
clone 3 – W	T	G	A	C	T	T	T	G	C	G	C	G	T	T	G	T	G	C	T	G	T	T	T	C	A	T	10 bp
clone 10 – W	T	G	A	C	T	T	T	G	C	G	C	G	T	T	G	T	G	C	T	G	T	T	T	C	A	T	10 bp
clone 16 – W	T	G	A	C	T	T	T	G	C	G	C	G	T	T	G	T	G	C	T	G	T	T	T	C	A	T	10 bp
clone 13 – W	T	G	A	C	T	T	T	G	C	G	C	G	T	T	G	T	G	C	T	G	T	T	T	C	A	T	10 bp
clone 1 – W	T	G	A	C	T	T	T	G	C	G	C	G	T	T	G	T	G	C	T	G	T	T	T	C	A	T	—
clone 14 – W	T	G	A	C	T	T	T	G	C	G	C	G	T	T	G	T	G	C	T	G	T	T	T	C	A	T	—
clone 7 – W	T	G	A	C	T	T	T	G	C	G	C	G	T	T	G	T	G	C	T	G	T	T	T	C	A	T	—
clone 6 – W	T	G	A	C	T	T	T	G	C	G	C	G	T	T	G	T	G	C	T	G	T	T	T	T	A	T	10 bp
clone 4 – W	T	G	A	C	T	T	T	G	C	G	C	G	T	T	G	T	G	C	T	G	T	T	T	T	A	T	10 bp
clone 12 – W	T	G	G	C	T	T	T	G	C	G	C	G	T	T	G	T	G	C	T	G	T	T	T	T	A	T	10 bp
clone 15 – EU/W	T	T	A	A	T	C	T	T	T	T	T	C	–	C	A	A	T	T ┃	T	G	T	T	T	T	A	T	10 bp
clone 8 – W/EU/W/E	T	G	G	C	T	T	T ┃	T	T	T	T	C	–	C	A	A	T	T ┃	C	G	C	T ┃	C	C	T	A	10 bp
clone 9 – W/E	T	G	G	C	T	T	T	G	C	G	C	G	T	T	G	T	G	C	C	G	C	T ┃	C	C	T	A	10 bp
clone 5 – W/E	T	G	A	C	T	T	C	G	C	G	C	G	T	T	G	T	G	C	C	G	C	T ┃	C	C	T	A	10 bp

Color coding in clones: black: consensus; 'Western': blue, 'Pyrenean': dark violet; 'Eastern': red, '*H. umbellatum*': pink, accession-specific: turquoise

y at 485 is shared with some 'Western' species, T is 'Pyrenean'-specific. Turquoise character states are accession-specific or shared with the other H. prenanthoides *samples. r in 190 is also shared with* H. gymnocerinthe*, but may be a homoplasy (see Additional file 3); for simplicity, it is marked here as accession-specific.*

Most clones show the 'Western' sequence which was also strongly dominating in direct sequencing. The last three clones are recombinant before the 3'-'interclade hybrid' signature (Table 1). Clone 8 shows three recombination points, the central 'Eastern' part contains four of the seven '*H. umbellatum*'-specific sites, the other three should occur downstream before the next recombination point, but were probably lost by intragenomic recombination events. Only clone 15 shows the 'Eastern' signature from the beginning; from <435 to >461, it is entirely missing in the sequenced clones, but presence of an ordinary 'Eastern' ribotype (i.e., without '*H. umbellatum*'-specific characters) is indicated by the polymorphisms at 446 and 461 in the direct sequence. Two unique polymorphisms at 44 and 195 (second peaks very small) are also not accounted for by these clones.

H. caesium	**46**	**187**	**194**	**197**	**232**	**233**	**244**	**252**	**256**	**262**	**281**	**307**	**326**	**349**	**368**	**425**	**426**	**436**	**446**	**459**	**461**	**478**	**479**	**485**	**486**	**488**
polymorphic	G/–	k	m	y	k	y	k	y	w	T/–	y	R	w	k	y	r	w	w	r	r	w	y	r	y	w	w
dominant peaks	G	G	C	T	G	C	G	C	T	T	T	R	T	G	C	A	T	T	G	G	T	T	A	C	A	T
clone 5 – W	–	G	C	T	G	C	G	C	T	T	T	G	T	G	C	A	T	T	G	G	T	T	A	T	A	T
clone 3 – W	G	G	C	T	G	C	G	C	T	T	T	G	T	G	C	A	T	T	G	G	T	T	A	T	A	T
clone 2 – W	G	G	C	T	G	C	G	C	T	T	T	G	A	G	C	A	T	A	G	G	T	T	A	T	A	T
clone 6 – W	G	G	C	T	G	C	G	C	T	T	T	G	T	G	C	A	T	T	G	G	T	T	A	C	A	T
clone 1 – W	G	G	C	T	G	T	G	C	T	T	T	G	T	G	C	A	T	T	G	G	T	T	A	C	A	T
clone 4 – EU	G	T	A	C	T	T	T	T	T	–	C	A	A	T ⋮	C	A	T	T ⋮	A	A	A	C	G	C	T	A

Color coding in clones: black: consensus; 'Western': blue, 'Pyrenean': dark violet; 'Eastern': red, '*H. umbellatum*': pink, accession-specific: turquoise

The indel at 46 is shared with H. villosum *1029. It is probably a homoplasy and therefore marked here as accession-specific. w at 256 is shared with* H. umbellatum *um.AM.1.*

Most clones show the 'Western' sequence which was dominant in direct sequencing; clone 4 shows mostly the '*H. umbellatum*' sequence. A at 326 in clone 2, T at 233 in clone 1, and C at 368 in clone 4 occur on the 'wrong' strands. At positions 425 and 426, '*H. umbellatum*' character states are evident only from direct sequencing, but missing in clone 4. These patterns may have been caused by partial gene conversion (233, 326) and/or by double recombinations (>349 to <446) in clone 4 (hatched vertical lines).

Additional file 2: Patterns of *ETS* recombination

All cloned accessions with partly recombinant sequences are shown. Results from direct sequencing (‘polymorphic’, ‘dominant peaks’) and some additional accessions with non-recombinant clones are for comparison. Positions correspond to Additional files 3 and 4; approximate recombination points inferred by visual inspection are indicated by bold vertical bars.

***H. pilosum* 1226/2**	**21**	**54**	**123**	**187**	**194**	**197**	**233**	**244**	**252**	**254**	**262**	**281**	**307**	**332**	**351**	**357**	**367**	**368**	**369**	**412**	**446**	**461**	**478**	**486**	**488**
polymorphic	r	R	Y	K	M	Y	Y	K	Y	S	T/–	Y	R	M	w	K	y	Y	k	y	R	W	Y	W	W
dominant peaks	G	R	Y	T	M	Y	Y	K	Y	S	T/–	Y	R	M	A	K	T	Y	G	C	R	W	C	W	W
clone 3 – Wy	G	A	C	G	C	T	C	G	C	C	T	T	G	C	A	T	T	C	G	C	G	T	T	A	T
clone 8 – Wy	G	A	C	G	C	T	C	G	C	C	T	T	G	C	A	T	T	C	G	C	G	T	T	A	T
clone 4 – Wy	G	A	C	G	C	T	C	G	C	C	T	T	G	C	A	T	T	C	G	C	G	T	T	A	T
clone 1 – Epo/Wy/Epo/W(y)	A	G ┃	C	G	C	T	C	G	C	C	T	T	G	C	A ┃	G	C	T	G ┃	C	G	T	T	A	T
clone 7 – Epo/Wy	G	G	T	T	A	C	T	T	T	G	– ┃	T	G	C	A	T	T	C	G	C	G	T	T	A	T
clone 2 – Wy/Epo/W(y)	G	A	C ┃	T	A	C	T	T	T	G	–	C	A	A	T	G	C	T	T ┃	C	G	T	T	A	T
clone 6 – Epo	G	G	T	T	A	C	T	T	T	G	–	C	A	A	A	G	C	T	G	T	A	A	C	T	A
clone 5 – Epo	G	G	T	T	A	C	T	T	T	G	–	C	A	A	A	G	C	T	G	T	A	A	C	T	A

Color coding in clones: black: consensus; ‘Western’: blue, ‘unknown Western 2’: light violet, ‘Eastern’: red, ‘*H. porrifolium*’: yellow, accession-specific: turquoise

A at 21 is unique for H. pilosum *1226/1. S at 254 is shared only with* H. villosum *1305 (see below). At this position T, C, and G and all three possible combinations of polymorphisms (S, Y, and K) occur in the dataset. C is here on the ‘Western’ strand, but in* H. olympicum *(see above), it is on the ‘Eastern’ strand suggesting that the character state C is homoplasious while the polymorphisms are not, but differentiate consistently between reasonable groups (see Additional file 3). C on the ‘Western’ ribotype at 254 is accompanied by Y at 123, M at 332, and K at 357. They are shared with the hybrids* H. villosum *1305,* H. gymnocephalum *(both accessions) and* H. plumulosum*. We refer to this pattern as ‘unknown Western 2’. y at 412 is shared with several* ‘H. porrifolium’ *species and hybrids and some* ‘H. umbellatum’ *clade accessions and hybrids; the latter are probably homoplasious, but ‘Eastern’ in any case.*

Three recombinant sequences occur of which two are probably double or triple recombinants. All polymorphic positions are reflected in the sequenced clones.

***H. villosum* 1305**	**123**	**187**	**194**	**197**	**233**	**244**	**252**	**254**	**262**	**281**	**307**	**332**	**351**	**357**	**367**	**368**	**373**	**412**	**446**	**461**	**478**	**486**	**488**
polymorphic	y	k	m	y	y	k	y	s	T/–	y	r	m	w	k	y	y	y	y	r	w	y	w	w
dominant peaks	T	T	A	C	T	T	T	G	–	C	A	A	T	G	C	T	C	C	A	A	C	T	A
clone 1 – Wy	C	G	C	T	C	G	C	C	T	T	G	C	A	T	T	C	C	C	G	T	T	A	T
clone 5 – Wy	C	G	C	T	C	G	C	C	T	T	G	C	A	T	T	C	C	C	G	T	T	A	T
clone 2 – Epo	T	T	A	C	T	T	T	G	–	C	A	A	T	G	C	T	C	C	A	A	C	T	A
clone 3 – Epo	T	T	A	C	T	T	T	G	–	C	A	A	T	G	C	T	C	C	A	A	C	T	A
clone 4 – Epo	T	T	A	C	T	T	T	G	–	C	A	A	T	G	C	T	C	C	A	A	C	T	A
clone 6 – Epo	T	T	A	C	T	T	T	G	–	C	A	C	T	G	C	T	C	C	A	A	C	T	A
clone 7 – Epo	T	T	A	C	T	T	T	G	–	C	A	A	T	G	C	T	C	C	A	T	C	T	A

Color coding in clones: black: consensus; ‘Western’: blue, ‘unknown Western 2’: light violet, ‘Eastern’: red, ‘*H. porrifolium*’: yellow

y at 373 is shared only with H. petrovae *(a Balkan endemic) and is probably a homoplasy (accession 1305 is from France). For more detailed explanation see the previous table.*

No obviously recombinant sequences occur in contrast to *H. pilosum* 1226/2 (above) which has the same inferred parentage except that probably ‘pure’ *H. villosum* was the maternal parent of *H. villosum* 1305 (the ‘*H. pilosum*’-specific polymorphism at position 21 is missing, and the plant was morphologically identified as *H. villosum*). Other differences to the above pattern are the dominance of the ‘Eastern’ (‘*H. porrifolium*’) ribotype and a lack of accession-specific polymorphism. The ‘*H. porrifolium*’ character T at 412 is only apparent from direct sequencing (y). Two ‘Western’ characters occur on ‘Eastern’ strands in different clones (position 332 in clone 6, and 461 in clone 7). Double recombinations directly before and after these positions (i.e. at intervals <50 bp) are probably less likely than either polymerase errors or some gene conversion activity.

Additional file 2: Patterns of *ETS* recombination

All cloned accessions with partly recombinant sequences are shown. Results from direct sequencing ('polymorphic', 'dominant peaks') and some additional accessions with non-recombinant clones are for comparison. Positions correspond to Additional files 3 and 4; approximate recombination points inferred by visual inspection are indicated by bold vertical bars.

***H. gymnocephalum* 1215**	**123**	**154**	**162**	**187**	**194**	**197**	**233**	**244**	**252**	**254**	**260**	**262**	**281**	**307**	**311**	**332**	**349**	**357**	**368**	**381**	**446**	**458**	**461**	**478**	**486**	**488**
polymorphic	Y	R	Y	K	M	Y	Y	K	Y	Y	W	T/–	y	R	Y	M	r	K	Y	r	R	Y	W	Y	W	W
dominant peaks	C	G	C	K	C	T	C	K	Y	Y	T	T/–	T	R	T	C	G	K	C	G	R	Y	T	Y	A	T
clone 2 – Wy	C	G	C	G	C	T	C	G	C	C	T	T	T	G	T	C	G	T	C	G	G	C	T	T	A	T
clone 3 – Wy	C	G	C	G	C	T	C	G	C	C	T	T	T	G	T	C	G	T	C	A	G	C	T	T	A	T
clone 1 – Ex	T	A	T	T	A	C	T	T	T	T	A	–	C	A	C	A	G	G	T	G	A	T	A	C	T	A

Color coding in clones: black: consensus; 'Western': blue, 'unknown Western 2': violet, 'Eastern': red, 'Balkan': orange, 'unknown Eastern' green, accession-specific: turquoise

r at 349 is accession-specific; the non-consensus character state was not retrieved by the sequenced clones.

No recombinant clones were found. The 'Western' ribotype is represented by the 'unknown Western 2' variant. In addition to 'ordinary' 'Eastern' character states in clone 1, A at 154 is shared with most 'pure' Balkan species (Figure 4). Four additional character states are found on the 'Eastern' ribotype (positions 162, 260, 311, and 458). They are shared with *H. plumulosum* and *H. heterogynum* and are referred to as the 'unknown Eastern' ribotype. The polymorphism pattern of both *H. gymnocephalum* samples is nearly identical except for a further 'Balkan' polymorphism of accession 1207 at position 344 and the lack of the accession-specific polymorphisms of accession 1215 shown here (349 and 381). Both ribotypes were present in equal amounts according to direct sequencing in both accessions.

H. heterogynum	**37**	**123**	**126**	**154**	**162**	**183**	**187**	**194**	**197**	**201**	**233**	**241**	**244**	**252**	**254**	**255**	**260**	**262**
polymorphic	T	T	y	R	Y	G	K	M	Y	y	Y	A	K	Y	T	k	W	T/–
dominant peaks	T	T	C	R	Y	G	K	M	Y	T	Y	A	K	Y	T	T	T	T/–
clone 3 – Wy	T	C	C	G	C	G	G	C	T	T	C	A	G	C	C	T	T	T
clone 4 – W	C	T	C	A	C	G	G	C	T	T	C	G	G	C	T	T	T	T
clone 5 – W	C	T	C	A	C	G	G	C	T	T	C	G	G	C	T	T	T	T
clone 2 – W	T	T	C	G	C	G	G	C	T	T	C	A	G	C	T	T	T	T
clone 1 – W	T	T	C	G	C	G	G	C	T	T	C	A	G	C	T	T	T	T
clone 7 – Ex	C	T	C	A	T	G	T	A	C	T	T	A	T	T	T	G	A	–
clone 8 – Ex	T	T	C	A	T	G	T	A	C	T	T	A	T	T	T	T	A	–
clone 10 – Ex	T	T	C	A	T	G	T	A	C	T	T	A	T	T	T	T	A	–
clone 9 – Ex	C	T	C	A	T	T	T	A	C	T	T	A	T	T	T	T	A	–
clone 6 – Ex	T	T	C	A	T	G	T	A	C	T	T	A	T	T	T	T	A	–
clone 11 – Ex	T	T	C	A	T	G	T	A	C	T	T	A	T	T	T	T	A	–

H. heterogynum	**268**	**269**	**280**	**281**	**302**	**307**	**311**	**332**	**337**	**357**	**368**	**426**	**446**	**458**	**461**	**478**	**479**	**485**	**486**	**488**
polymorphic	r	w	r	Y	R	R	Y	A	w	G	Y	W	R	Y	W	Y	r	C	W	W
dominant peaks	A	A	G	Y	G	R	Y	A	T	G	Y	T	R	Y	W	Y	A	C	W	W
clone 3 – Wy	A	A	G	T	G	G	T	C	T	T	C	T	G	T	T	T	A	C	A	T
clone 4 – W	A	A	G	T	G	G	T	A	T	G	C	T	G	C	T	T	A	T	A	T
clone 5 – W	A	A	G	T	G	G	T	A	T	G	C	T	G	C	T	T	A	T	A	T
clone 2 – W	A	A	G	T	A	G	T	A	T	G	C	A	G	C	T	T	A	C	A	T
clone 1 – W	A	A	G	T	A	A	T	A	T	G	C	A	G	C	T	T	A	C	A	T
clone 7 – Ex	G	A	A	C	G	A	C	A	T	G	T	T	A	T	A	C	G	C	T	A
clone 8 – Ex	G	A	G	C	G	A	C	A	T	G	T	T	A	T	A	C	G	C	T	A
clone 10 – Ex	A	A	G	C	G	A	C	A	T	G	T	T	A	T	A	C	G	C	T	A
clone 9 – Ex	A	A	G	C	G	A	C	A	T	G	T	T	A	T	A	C	A	C	T	A
clone 6 – Ex	A	A	G	C	G	A	C	A	T	G	T	T	A	T	A	C	A	C	T	A
clone 11 – Ex	A	A	G	C	G	A	C	A	A	G	T	T	A	T	A	C	A	C	T	A

Color coding in clones: black: consensus; 'Western': blue, 'unknown Western 2': violet, 'Eastern': red, 'Balkan': orange, '*H. umbellatum*': pink, 'unknown Eastern' green, accession-specific: turquoise

Accession-specific character states at 37, 183, and 241 as well as the 'unknown Western 2' patterns were not found in direct sequencing (repeated in this case) indicating that they are present in less than 5% of all copies present in the genome. In contrast, the alternative character states of polymorphisms at 126, 201, and 269 are not represented by these clones.

About half of the clones show 'Western' and 'Eastern' ribotypes, respectively, reflecting the results from direct sequencing. No obvious recombinants occurred among these clones. The 'Eastern' ribotype corresponds to the 'unknown Eastern' variant (see also *H. gymnocephalum* and *H. plumulosum*). One of the '*H. umbellatum*'-specific character states (at 479) is present on part of the 'Eastern' clones, a second one (at 426) on two 'Western' sequences. Five further '*H. umbellatum*'-specific character states were neither present in the direct sequences nor among the clones suggesting that they were lost from the genome. Nevertheless, contribution of the '*H. umbellatum*' clade is apparent from the chloroplast haplotype of *H. heterogynum*. The two '*H. umbellatum*'-specific character states that could be retrieved may have been retained only because they occur on other than typical '*H. umbellatum*' ribotypes. A possible explanation is gene conversion, which might also to have been responsible for A at 154 in clones 4 and 5, A at 307 in clone 1, and T at 458 in clone 3; all of these represent single character states occurring on the 'wrong' strand.

Additional file 2: Patterns of *ETS* recombination

All cloned accessions with partly recombinant sequences are shown. Results from direct sequencing ('polymorphic', 'dominant peaks') and some additional accessions with non-recombinant clones are for comparison. Positions correspond to Additional files 3 and 4; approximate recombination points inferred by visual inspection are indicated by bold vertical bars.

H. plumulosum	46	123	154	162	171	186	187	194	197	201	230	233	244	251	252	254	260	262	281	307	311	323	325	332	357	368	416	431	434	445	446	458	461	478	486	488	520
polymorphic	k	y	r	y	r	y	k	m	y	w	y	y	k	k	y	y	W	T/–	y	r	Y	y	r	m	k	y	r	k	r	y	r	Y	w	y	w	w	y
dominant peaks	G	T	A	T	A	C	T	A	C	T	T	T	T	T	T	T	W	–	C	A	Y	C	G	A	G	T	A	G	A	C	A	Y	A	C	T	A	T
clone 1 – W	T	T	G	C	G	C	G	C	T	T	T	C	G	T	C	C	T	T	T	G	T	C	G	A	G	C	A	G	A	C	G	C	T	T	A	T	T
clone 3 – W/Wy	G	T	G	C	G	C	G	C	T	T	T	C	G	T	C	│C	T	T	T	G	T	C	G	C	T	C	A	G	A	C	G	C	T	T	A	T	T
clone 4 – W/Wy	G	T	G	C	G	C	G	C	T	T	T	C	G	T	C	│C	T	T	T	G	T	C	G	C	T	C	A	G	A	C	G	C	T	T	A	T	T
clone 7 – Wy	G	C	G	C	G	C	G	C	T	T	T	C	G	T	C	C	T	T	T	G	T	C	G	C	T	C	A	G	A	C	G	C	T	T	A	T	T
clone 8 – Wy	G	C	G	C	G	C	G	C	T	T	T	C	G	T	C	C	T	T	T	G	T	C	G	C	T	C	A	G	A	C	G	C	T	T	A	T	T
clone 2 – Wy/Ex	G	C	G	C	G	C	G	C	T	T	T	C	G	T	C	C	T	T	T	G	T	C	G	C	G	C	A	G	A	C	│A	T	A	C	T	A	T
clone 5 – Wy/Ex	G	C	G	C	G	C	G	C	T	T	T	C	G	T	C	C	T	T	T	G	T	C	G	C	T	C	A	G	A	C	│A	T	A	C	T	A	T
clone 6 – Wy/Ex	G	C	G	C	G	C	G	C	T	T	T	│T	T	T	T	T	A	T	C	A	C	C	G	A	G	T	A	G	A	T	A	T	A	C	T	A	T
clone 9 – Ex	G	T	A	T	A	C	T	A	C	T	T	T	T	T	T	T	A	–	C	A	C	T	G	A	G	T	A	G	A	T	A	T	A	C	T	A	C
clone 11 – Ex	T	T	A	T	A	C	T	A	C	T	T	T	T	T	T	T	A	–	C	A	C	T	G	A	G	T	A	G	A	T	A	T	A	C	T	A	T
clone 10 – Ex	G	T	A	T	A	C	T	A	C	T	T	T	T	T	T	T	A	–	C	A	C	T	G	A	G	T	A	G	A	T	A	T	A	C	T	A	T
clone 13 – Ex	G	T	A	T	A	C	T	A	C	T	T	T	T	T	T	T	A	–	C	A	C	T	G	A	G	T	A	G	A	T	A	T	A	C	T	A	T
clone 14 – Ex	G	T	A	T	A	C	T	A	C	T	T	T	T	T	T	T	A	–	C	A	C	T	G	A	G	T	A	G	A	T	A	T	A	C	T	A	T
clone 15 – Ex	G	T	A	T	A	C	T	A	C	T	T	T	T	T	T	T	A	–	C	A	C	T	G	A	G	T	A	G	A	T	A	T	A	C	T	A	T
clone 12 – Ex/E	G	T	A	T	A	C	T	A	C	T	T	T	T	T	T	T	A	–	C	A	│T	C	A	A	G	T	G	T	G	C	A	C	A	C	T	A	C

Color coding in clones: black: consensus; 'Western': blue, 'unknown Western 2': violet, 'Eastern': red, 'Balkan': orange, 'unknown Eastern' green, accession-specific: turquoise

k at 46 is only shared with H. naegelianum, *another Balkan species. It occurs on the 'wrong' ribotype in clone 1. r at 171 and y at 323 are homoplasious (Additional file 3) and are marked here as accession-specific. Both have accumulated on the same major ribotype. C at 254 is here on the 'Western' strands like in* H. pilosum *1226/2,* H. villosum *1305, and* H. heterogynum, *reflecting the 'unknown Western 2' ribotype. T is the consensus at this position. In contrast, in* H. olympicum, *C is on the 'Eastern' strand and is shared with 'pure' Balkan species (see above).*

Clone 1 shows an ordinary 'Western' sequence apart from positions 46 and 254. Clones 3 and 4 may be recombinants between genuine 'Western' and 'unknown Western 2' variants. The putative recombination point could be anywhere between >123 and <254 in this case. Clones 7 and 8 show all character states identifying the 'unknown Western 2' type. Clones 2, 5, and 6 are recombinant between this type and an 'Eastern' sequence, clone 6 with a different recombination point. Clones 9–15 are entirely 'Eastern'. All represent the 'unknown Eastern' lineage except clone 12 which is recombinant between this variant and an almost 'pure' 'Eastern' ribotype. Its 3'-half also contains several accession-specific and one 'Balkan' character state. All accession-specific substitutions (as far as retrieved) are confined to 'Eastern' sequences. For four further accession-specific polymorphisms (at 186, 201, 230, and 251) in the 5'-part of the *ETS*, the alternative nucleotide was not retrieved by these clones. Some gene conversion may have occurred as suggested by single character states occurring on the 'wrong' variant: T at 46 and C at 254 in clone 1; G at 357 in clone 2; T at the indel position and C at 323 in clone 6; and C at 520 in clone 9.

The 'Eastern' sequence was strongly dominating in direct sequencing, but no 'ordinary' 'Eastern' ribotype was found among these clones apart from the 3'-part in clone 12. Interestingly, at the four positions reflecting the 'unknown Eastern' polymorphisms, the consensus character states (here only found on 'Western' ribotypes) showed a markedly stronger signal than in the rest of the sequence. This indicates that only part of the 'Eastern' sequences can possibly show the 'unknown Eastern' ribotype, because the ordinary 'Eastern' type also has the consensus character states at these positions. If the 'unknown Eastern' variant was the only 'Eastern' ribotype, the proportions of 'Western' and 'Eastern' character states should be the same along the whole sequence, not differ only at these diagnostic sites. In order to retrieve a complete 'Eastern' sequence, we did restriction digests of further clones: An *Eco*RI digest affecting pos. 194 and 197 distinguishes between 'Eastern' and 'Western' ribotypes; among the 'Eastern' ones, a *Hha*I digest affecting pos. 154 can distinguish 'Eastern' ribotypes from those with a 'Balkanian' A, and a *Tha*I digest affecting position 162 can distinguish the 'unknown Eastern' from ordinary 'Eastern' variants. Nine additional 'Eastern' clones preselected by *Eco*RI digests had A at 154 and T at 162 after the two subsequent digests, i.e., they also contained the 'unknown Eastern' ribotype and were therefore not sequenced. However, relative peak heights in direct sequencing, the 3'-part of clone 12, and four missing accession-specific character states in the 5'-half of the cloned sequences indicate that the 'ordinary' (or rather '*H. plumulosum*'-specific because of many unique intra-individual polymorphisms) 'Eastern' ribotype must be present and that the cloned sequences were not entirely representative in this case.

Additional file 2: Patterns of *ETS* recombination

All cloned accessions with partly recombinant sequences are shown. Results from direct sequencing ('polymorphic', 'dominant peaks') and some additional accessions with non-recombinant clones are for comparison. Positions correspond to Additional files 3 and 4; approximate recombination points inferred by visual inspection are indicated by bold vertical bars.

H. kittanae	**49**	**154**	**189**	**254**	**256**	**311**	**317**	**344**	**378**	**385**	**390**	**445**	**468**	**501**	**514**	**515**
polymorphic	y	r	y	y	y	k	y	y	T	y	r	y	r	y	s	m
dominant peaks	C	G	C	C	T	G	C	C	T	C	A	C	G	C	C	C
clone 1 – EB	C	G	C	C	T	G	T	C	T	C	A	C	G	C	C	A
clone 4 – EB	C	G	C	C	T	G	T	C	T	C	A	C	G	C	C	A
clone 8 – EB	C	G	C	C	T	G	T	C	T	C	A	C	G	C	C	A
clone 7 – EB	C	G	C	C	T	G	C	C	C	C	A	C	G	C	C	C
clone 3 – EB	C	G	C	C	T	G	C	C	C	C	A	T	A	T	G	C
clone 5 – EB	C	G	C	C	T	G	C	C	T	C	A	C	G	T	G	C
clone 2 – EB	C	G	C	C	T	G	C	C	T	C	A	C	G	C	C	C
clone 9 – EB	C	G	C	T	C	T	C	T	T	T	A	C	G	C	C	A
clone 10 – EB	T	A	T	T	C	T	C	T	T	T	G	C	A	C	C	C

Color coding in clones: black: consensus; 'Balkan': orange, accession-specific: turquoise

T at 317 is most probably a homoplasy as it occurs only in H. kittanae *and in many 'Western' species without any further evidence for a 'Western' contribution. It is therefore marked here as accession-specific. C at 378 is unique at this position for the whole dataset and was not reflected by a polymorphism in the direct sequence. It could be a polymerase error in an earlier round of the PCR, but was not corrected because it occurred in two clones. It is also marked as accession-specific.*

Exclusively 'Eastern' patterns were found, most of them are shared with various species from the Balkans in different combinations (Figure 4). Only three out of nine clones are identical (clones 1, 4, and 8). A scattered distribution of derived character states on different strands is apparent. These patterns are too inconclusive to infer potential recombination. All non-consensus character states were present in amounts of only 10–20% of the total signal in direct sequencing. All were retrieved by these clones. Matching patterns consistently occurring in other species are missing (see also Figure 4).
