# Supplementary material for: Intra-individual polymorphism in diploid and apomictic polyploid hawkweeds (Hieracium, Lactuceae, Asteraceae): disentangling phylogenetic signal, reticulation, and noise
Source: BMC Evol Biol. 2009 Sep 22;9:239. doi: 10.1186/1471-2148-9-239 (PMC2759941; doi:10.1186/1471-2148-9-239)
Supplement: Additional file 3 — Summary of intra-individual polymorphisms. All intra-individual polymorphisms are listed according to their position in the alignment along with the accessions in which they occur, discriminating additive, unique, and shared variation. Among the shared polymorphisms, homoplasious and informative ones are identified according to specified criteria. [file 1471-2148-9-239-S3.PDF]

Additional file 3:
Summary of intra-individual polymorphisms

Species abbreviations:

alpinum: alp
amplexicaule: amp
bifidum: bif
bracteolatum: bra
bupleuroides: bup
caesium: cae
canadense: cde
candidum: cnd
cerinthoides: cer
cordifolium: cor
eriophorum: eri
glaucum: gla
gouani: gou
gymnocephalum: gcl
gymnocerinthe: gci
heterogynum: het
humile: hum
kittanae: kit
lachenalii: lch
laevigatum: lae
lawsonii: law
lucidum: luc
mixtum: mix
murorum: mur
naegelianum: nae
olympicum: oly
pannosum: pan
petrovae: pet
pictum: pic
pilosum: pil
plumulosum: plu
pojoritense: poi
porrifolium: por
prenanthoides: pre
racemosum: rac
ramondii: ram
recoderi: rec
sabaudum: sab
schmidtii: sch
sparsum: spa
stelligerum: ste
tomentosum: tom
transylvanicum: tra
umbellatum: umb
villosum: vil
virosum: vir

Legend:

Position in the alignment, character states (IUPAC codes: Y = C or T; R = A or G; M = C or A; W = A or T; K = G or T; S = G or C; H = A, C, or T; D = A, G or T) and accessions showing the respective intra-individual polymorphisms in direct sequencing are given. Positions marked with an asterisk (*) have more than one kind of polymorphism.

Unique polymorphisms (for single accessions or species) are marked in orange. If all accessions of a given species shared the same polymorphism, this is indicated by (2) or (3) after the species abbreviation; accession labels are given if needed for distinction (for accession details, see Additional file 5: Species/accessions, their origin, cytotype, *ETS* and cpDNA features).

Fully or partly homoplasious polymorphisms are indicated in blue. Criteria for assuming homoplasy (usually, more than one applied) are: (i) accessions for which recent or past overlap of distribution areas is highly unlikely (geography), (ii) accessions belonging to divergent clades or hybrids with certainly different origin (phylogeny), or (iii) there was no further evidence for these particular relationships (singularity). Blue labeling does not necessarily exclude the presence of phylogenetic signal or evidence of hybridization (e.g., in case of single ‘outliers’ among otherwise supported groups of accessions). Such groups of presumably related accessions are separated by a slash (/).

If homoplasy could not be excluded according to these criteria, the position is given in brown color. These sites are most likely to exhibit meaningful (informative) shared polymorphisms.

Character additivity (alternative character states present as monomorphic characters in the dataset) is indicated by ‘+’. In case polymorphisms reflect informative substitutions of particular subclades or lineages, their character state is given in brackets; the alternative character state of the polymorphism is the consensus. In most cases, the second character state is missing in the dataset. Additive characters occur in all categories (Fig. 1).

Some frequently occurring groups of accessions are abbreviated: ‘unknown Western 1’ = luc, pre1161, bra, rac, sab, and oly; *um* = all accessions of umb, eri, cde, and vir (‘*H. umbellatum*’ group); and ‘interclade hybrids’ = amp, bra, cae, gla, gou, gcl(2), het, lae, mix, oly, pil1226/2, plu, pre1187, rac, sab, and vil1305; less obvious interclade hybrid accessions (lch, pre1252, pre1161) are indicated separately.

At position 254, three kinds of polymorphism occur (K, S, Y) reflecting all combinations of the different character states occurring in the dataset (C, T, G). Y at this position is caused by the C occurring either on the ‘Eastern’ ribotype (spa, kit, oly) or on the ‘Western’ one (plu, gcl). This polymorphism is not marked as homoplasious here because it could not be inferred from the criteria specified above, but only from cloned sequences (see also Additional file 2: Patterns of *ETS* recombination). S at this position is also composed of a ‘Western’ C, but in combination with G situated on the ‘Eastern’ (‘*H. porrifolium*’) sequence. C on the ‘Western’ ribotype reflects the ‘unknown Western 2’ sequence.

Pos.	N	accessions
2	Y	pan
4	Y	luc
13	Y	vir1238
20	K	alp.Ukr
21	R	pil1226/2 (pil1226/1: A) +
35	Y	eri(2)
43	K	cor
44	Y	pre(1252,1187) / nae – geogr., phylogeny, singularity
45	S	spa(2) / mix – geography, phylogeny, singularity
46*	-/G	cae / vil1029 – geography, phylogeny, singularity
46*	K	nae, plu
49	Y	vil1029 / kit, pan – geography, phylogeny, singularity
54	R	pil1226/2
56	R	vil1029
62	Y	mur
64	Y	cnd, amp
68	Y	lae
69	Y	sch
78	Y	luc
80	Y	lae
92	Y	tra1077
112	Y	nae
119	K	eri1222
120	W	poi (alp: A) +
123*	W	poi (alp: A) +
123*	Y	vil1305, pil1226/2, gcl(2), plu (‘unknown Western 2’)
126	Y	het
130	Y	bra
137*	K	tom
137*	Y	poi / mur – phylogeny, singularity
139	R	cde
145	Y	tra.Boa
154	R	het, gcl(2), plu, kit (pan, pet and nae: A) +
162	Y	het, gcl(2), plu (‘unknown Eastern’) / mix / alp.Boa – geography, singularity
167*	K	spa1251
167*	Y	alp.Boa
169	W	‘unknown Western 1’
171	R	law, rec, cer, cor, gci, cnd, gou, amp (ram: G) + / plu / por – geography, phylogeny, singularity
175	Y	lch
176	Y	lch
180	R	‘unknown Western 1’ / poi (alp: A) + – phylogeny, singularity
181	Y	sch
183	K	mix
186	Y	plu
187	K	interclade hybrids +
188*	R	bif
188*	M	spa1251
189	Y	kit, pan
190	R	gci / pre(3) – singularity
194*	M	interclade hybrids + / nae – singularity
194*	W	por, bup(2)
194*	H	gla +
195	Y	alp(2) / pre1187 – geography, phylogeny, singularity
196-197	--/TY	‘unknown Western 1’
197	Y	interclade hybrids except ‘unknown Western 1’ +
201*	Y	het
201*	W	plu
202	K	gou, mix
205	Y	pre1187
206	W	tra.Boa

Pos.	N	accessions
207	S	tom
209	S	ram
213	R	cde
214	Y	poi
215	R	oly
225	Y	lae, cde, vir / pet – phylogeny, singularity
230	Y	plu
231	K	vir1238
232*	K	lae, pre1187, bra, rac, sab, cae, bup1033, poi (*um*:T) +
232*	S	luc
232*	R	rec
233	Y	interclade hybrids + / por – singularity
240	W	law
241	R	luc
244	K	pic1307 / interclade hybrids + – singularity
246	Y	pre1252 / pan – geography, phylogeny, singularity
251	K	plu
252	Y	lch, pre1161, interclade hybrids +
253*	S	pre1187, cde, lae
253*	K	bif
254*	K	vil1029, bup1033, gla (por, bup1212, pil1226/1: G) +
254*	S	vil1305, pil1226/2 (‘unknown Western 2’)
254*	Y	spa(2), kit, oly (Balkan), plu, gcl(2) (‘unknown Western 2’)
255*	K	het
255*	Y	poi
256*	W	um.AM.1, cae
256*	Y	kit, pan, nae
258	R	luc
259	M	cer, cor, gci, cnd, amp, gou (rec, ram, law: A) +
260	W	pic1067 / het, plu, gcl(2) (‘unknown Eastern’) – geography, singularity
262	-/T	interclade hybrids +
263	-/T	ste
264	R	tra1077
268	R	lae, het / nae – phylogeny, singularity
269*	M	amp
269*	W	het
270	W	tom
271	K	pet
275	Y	bup(2) / nae – geography, singularity
276*	W	oly
276*	Y	pet
277*	Y	tra1077
277*	W	por
280	R	het / amp – geography, phylogeny, singularity
281	Y	interclade hybrids + / por / poi, alp.Boa – singularity
284	Y	mur
292	Y	pan (pet: C) +
297	R	eri(2)
298	Y	mix
301	W	umb1021
302	R	ste, mur, bif, lch / het / pan – geography, singularity
303	R	alp.Ukr / bra – phylogeny, singularity
304	R	alp.Ukr
307	R	interclade hybrids, lch +
311*	K	sch / spa(2), kit – geography, phylogeny, singularity
311*	Y	het, plu, gcl(2) (‘unknown Eastern’)
312	S	luc
314	R	bif
317*	Y	luc, law, ram, rec, cer, gci, cnd, gou, amp / kit – geography, phylogeny, singularity
317*	M	umb1021

Pos.	N	accessions
320	K	umb1021
323	Y	vil1029 / plu – geography, singularity
325*	R	plu
325*	K	tra1077
326*	W	pre1187, bra, rac, cae, sab, lae, bup1033, poi (*um*: A) +
326*	K	pic1067
327	R	sch / vir1238 – geography, phylogeny, singularity
332*	M	luc / pil1226/2, vil1305, gcl(2), plu (‘unknown Western 2’) – geography, phylogeny
332*	R	vir.1
336	R	hum1188
337	W	sch / het
339	Y	spa.sst.2
340*	K	alp.Boa, poi (alp.Ukr: T) +
340*	R	gla
341*	W	nae
341*	Y	mix
343	R	amp / vir.1 – geography, singularity
344	Y	gci, mix / kit, pan, pet, gcl1207 / poi – singularity
345	R	cor
346*	M	lch
346*	Y	umb(2), eri(2), cde, rac, sab, poi
349*	K	pre1187, bra, rac, sab, lae, cae, bup1033, poi, vir(2) (*um* except vir(2): T) +
349*	R	gcl1215
351	W	vil(2), pil1226/2
352	W	pic1067
355	W	poi
357	K	pil1226/2, vil1305, plu, gcl(2) (‘unknown Western 2’)
358	K	bif / ‘unknown Western 1’ – singularity
364	Y	mur
367	Y	amp / gla, pil1226/2, vil(2), bup1033 (por, bup1212, pil 1226/1: C) + – phylogeny, singularity
368	Y	interclade hybrids +
369*	R	cer, gci, gou
369*	K	vil1029, pil1226/2
370	R	poi
373	Y	pet / vil1305 – phylogeny, singularity
374	R	spa.sst.2
375	Y	bup(2), por, gla / oly – phylogeny, singularity
376	Y	tra1077, law, ram, rec, cer, gci, cnd, gou, amp
381	R	gcl1215
385	Y	spa(2), kit, oly, het
386	Y	nae
390	R	spa(2), kit, oly
393	R	eri(2), um.AM.1, poi, sab
394	Y	nae
395	K	gla, bif, hum1064
396	R	tom
399	M	poi
403-404	--/TG	eri1221
406	Y	luc
412*	Y	pil(2), vil(2), bup1212 / eri(2), um.AM.1, sab, poi – singularity
412*	M	nae
413	Y	bif
414	R	pan / amp – geography, singularity
416	R	plu
419	K	gla
424	Y	vil1029
425*	R	amp / cae, lae, sab, rac, bra, poi (*um*: G) + – phylogeny, singularity
425*	W	bup1212
425*	D	bup1033 +
426*	W	ste / cae, lae, sab, rac, bra, het, poi (*um*: A) + – phylogeny, singularity
426*	K	bup1212
426*	D	bup1033 +
431	K	plu
434	R	spa(2), pan, nae, plu, oly (kit: G) +
435	Y	pre(3)
436	W	cae
440	K	tra.Boa
445	Y	tom, hum1064 / kit, plu – geography, phylogeny, singularity
446	R	interclade hybrids +
448	Y	pre(3)
449	S	bra
452	Y	tra1077, bif, ste (tra.Boa: T) +
456	S	bif
458	Y	gcl(2), het, plu (‘unknown Eastern’) / lch, tra.Boa – phylogeny, singularity
459	R	lae, bra, rac, sab, cae, bup1033, poi (*um*: A) +
461*	W	interclade hybrids +
461*	Y	tra.Boa
463	M	spa(2), alp.Boa
465*	M	nae
465*	S	cde
468	R	spa(2), kit, oly
470	Y	bif
475*	R	pet, pan
475*	K	cnd
478	Y	sch, rec / pre(3), interclade hybrids + – singularity
479	R	oly / het, lae, cae, bup1033, poi (*um*, bra, rac, sab: G) + – phylogeny, singularity
480	Y	cor
481	Y	lae
482*	R	pan, pet
482*	W	poi
485*	Y	tom, lch, mur, pic1067, cer, cor, gci, cnd, gou, pre1187, mix, amp, lae, cae, het (law, ram, rec: T) +
485*	M	bra
486	W	pre(3), interclade hybrids +
487	Y	vir1238
488	W	pre(3), interclade hybrids +
489	K	luc
491	Y	lae
494	R	um.AM.1
496-505	±10bp	pre1187
500	R	vir.1
501	Y	pan, kit, nae
502	M	sch
503	Y	pan, spa(2) (pet: C) +
505	R	poi
508-512	±5bp	cnd
514	S	kit, oly, spa(2)
515*	Y	tra1077 (tra.Boa: T) +
515*	M	kit / eri(2) / mix – geography, phylogeny, singularity
517*	K	mix
517*	S	eri(2)
518	K	poi
519	K	poi
520	Y	plu
527	R	sch
529	R	um1021
564	Y	cor
