# Supplementary material for: Intra-individual polymorphism in diploid and apomictic polyploid hawkweeds (Hieracium, Lactuceae, Asteraceae): disentangling phylogenetic signal, reticulation, and noise
Source: BMC Evol Biol. 2009 Sep 22;9:239. doi: 10.1186/1471-2148-9-239 (PMC2759941; doi:10.1186/1471-2148-9-239)
Supplement: Additional file 5 — Species/accessions, their origin, cytotype, ETS and cpDNA features. This table summarizes the information about individual accessions. [file 1471-2148-9-239-S5.PDF]

Additional file 5: Species/accessions, their origin, cytotype, *ETS* and cpDNA features

Species	Access.	Origin	Ploidy[1]	DNA content 2C [pg][1]	Inferred origin[2] (*ETS*)	cpDNA[3] (*trnT-L*)	Remarks
H. alpinum	alp.Ukr	Ukraine	2x	7.9	EA	EA	
	alp.Boa.2	Romania	2x	n.d.	EA	EA	
H. amplexicaule	1050/1	Austria	3x	10.8	WP-E	dWP1	'Western' *ETS* dominating
H. bifidum	1213/2	Slovakia	3x	10.7	W	W	
H. bracteolatum	1240/2	N Greece	3x	12.4	Wx-EU	EU	
H. bupleuroides	1212/2	Slovakia	3x	11.7	Epo	Epo	
	1033/3	Slovakia	3x	12.0	EU-Epo	EU	
H. caesium	**1231**	Sweden	4x	14.6	W-EU	W	'Western' *ETS* dominating
H. canadense	canad	Canada	3x	12.3	EU	EU	
H. candidum	1197/3	Spain	3x	n.d.	WP-W	WP2	
H. cerinthoides	1176/2	Spain	3x	10.8	WP-W	WP2	
H. cordifolium	1177/5	Andorra	2x	7.2	WP-W	dWP2	
H. eriophorum	1221/1	France	2x	8.5	EU	EU	
	1222/2	France	2x*	n.d.	EU	EU	
H. glaucum	1230/3	Slovenia	3x	11.4	W-Epo	Epo	
H. gouani	1171/4	Spain	2x	7.1	WP-E	WP1	'Western' *ETS* dominating
H. gymnocephalum	**1215**/1	Albania	2x	8.4	Wy-Ex	dO	
	1207/2	Montenegro	3x	n.d.	Wy-Ex	dO	
H. gymnocerinthe	1172/4	Spain	3x	10.7	WP-W	WP1	
H. heterogynum	**1250**/2	Montenegro	3x	12.5	W-Wy-Ex-EU	EU	partial *ETS* additivity for EU
H. humile	1064/2	Austria	4x	14.4	W	W	
	1188/2	France	3x	10.6	W	W	
H. kittanae	**1228**/2	Bulgaria	2x	8.4	EB	EB	
H. lachenalii	1160/2	Czechia	3x	11.3	W	EU	'Western' *ETS*, cpDNA captured from EU
H. laevigatum	1031/11	Czechia	3x	12.2	W-EU	EU	
H. lawsonii	1175/1	France	3x	10.9	WP	WP2	
H. lucidum	H. lucidum	Sicily	2x*	n.d.	W-Wx	W	
H. mixtum	**H. mixtum**	Germany	3x*	n.d.	W-E	unique	hybrid *ETS* dominating (Table 1)
H. murorum	875/1	Czechia	3x	10.7	W	W	
H. naegelianum	1208/2	Montenegro	3x	10.9	EB	unique	
H. olympicum	**1206**/3	Bulgaria	3x	12.1	Wx-EB	O	
H. pannosum	1205/1	Bulgaria	3x	11.7	EB	EB	
H. petrovae	1229	Bulgaria	2x	7.9	EB	EB	
H. pictum	1067/4	France	3x	10.6	W	W	
	1307/5	France	3x	10.7	W	W	
H. pilosum	1226/1	Slovenia	3x	11.6	Epo	Epo	
	**1226/2**	Slovenia	3x	11.8	Wy-Epo	Epo	
H. plumulosum	**1218**/2	Montenegro	2x	8.6	W-Wy-Ex-E	unique	'Eastern' *ETS* dominating (Table 1), multiple introgressions probable
H. pojoritense	poi.Rom.1	Romania	2x	n.d.	EA-EU	EA	'*H. alpinum*' *ETS* dominating
H. porrifolium	1052/9	Austria	2x	7.8	Epo	Epo	
H. prenanthoides	1252	France	2x	7.1	W-E	W	'Western' *ETS*, 3'-end additive (Table 1)
	1161/2	Poland	3x	10.9	W-E-Wx	dW	'unknown Western 1' *ETS*, 3'-end additive (Table 1)
	**1187**/1	Andorra	3x	11.5	W-E-EU	W	'Western' *ETS* dominating, 3'-end additive (Table 1), partial *ETS* additivity for EU
H. racemosum	874	Czechia	3x	12.5	Wx-EU	EU	
H. ramondii	1173/3	Andorra	3x	10.7	WP	WP1	
H. recoderi	1174/4	Spain	2x	7.1	WP	WP1	
H. sabaudum	1098/2	Germany	3x	12.6	Wx-EU	EU	
H. schmidtii	1025/3	Czechia	3x	10.7	W	W	
H. sparsum	1251/1	Bulgaria	2x	8.1	EB	dEA	cpDNA captured from EA
	spa.sst.2	Bulgaria	2x*	n.d.	EB	dEA	cpDNA captured from EA
H. stelligerum	1233/1	S France	2x	7.0	W	W	
H. tomentosum	1066/8	France	2x	7.5	W	W	
H. transylvanicum	tra.Boa	Romania	2x	8.3	W	unique	genome size and distribution may suggest cryptic 'Eastern' origin
	1077/7	Ukraine	2x	8.5	W	unique	genome size and distribution may suggest cryptic 'Eastern' origin
H. umbellatum	1021/1	Poland	2x	8.3	EU	EU	
	um.AM.1	Germany	2x	n.d.	EU	EU	
H. villosum	1029/1	Slovakia	4x	15.7	Epo	dEpo	
	**1305**/3	France	3x	11.6	Wy-Epo	Epo	'Eastern' *ETS* dominating
H. virosum	1238/1	Russia	3x	13.0	EU	EU	
	vir.1	Russia	3x	13.0	EU	unique	

[1] Chromosome counts and DNA content determined by flow cytometry, data adopted from [46].

[2] Apart from 'Eastern' (E) and 'Western' (W) ribotypes that could not be further differentiated, the following subgroups are indicated (corresponding to Figures 2 and 5): 'Pyrenean' (WP), 'unknown Western 1' (Wx), 'unknown Western 2' (Wy), '*H. alpinum*' (EA), '*H. umbellatum*' (EU), '*H. porrifolium*' (Epo), 'Balkan' (EB), 'unknown Eastern' (Ex).

[3] Chloroplast haplotypes (see Figure 3): WP1 and WP2 are different haplotypes of Pyrenean species; the '*H. olympicum*' haplotype is specified as "O". Haplotypes derived from other haplotypes / haplotype groups are given as, e.g., dWP1.

Asterisks after ploidy levels indicate chromosome counts reported for that species (in the strict sense), but not available for the particular accession.

The German accession of the Pyrenean species *H. mixtum* is from a recently described neophytic population (Bräutigam S, Gottschlich G, Hänel K. 2007. *Hieracium mixtum* FROEL. – ein für Deutschland neuer Neophyt. Kochia 2: 25-30).

For accessions marked in boldface, cloned sequences are shown in Additional file 2: Patterns of *ETS* recombination.
