# Supplementary figures and images for: Intra-individual polymorphism in diploid and apomictic polyploid hawkweeds (Hieracium, Lactuceae, Asteraceae): disentangling phylogenetic signal, reticulation, and noise
Source: BMC Evol Biol. 2009 Sep 22;9:239. doi: 10.1186/1471-2148-9-239 (PMC2759941; doi:10.1186/1471-2148-9-239)

# Additional file 6 - *ETS* phylogeny with ribotypes present only in hybrids

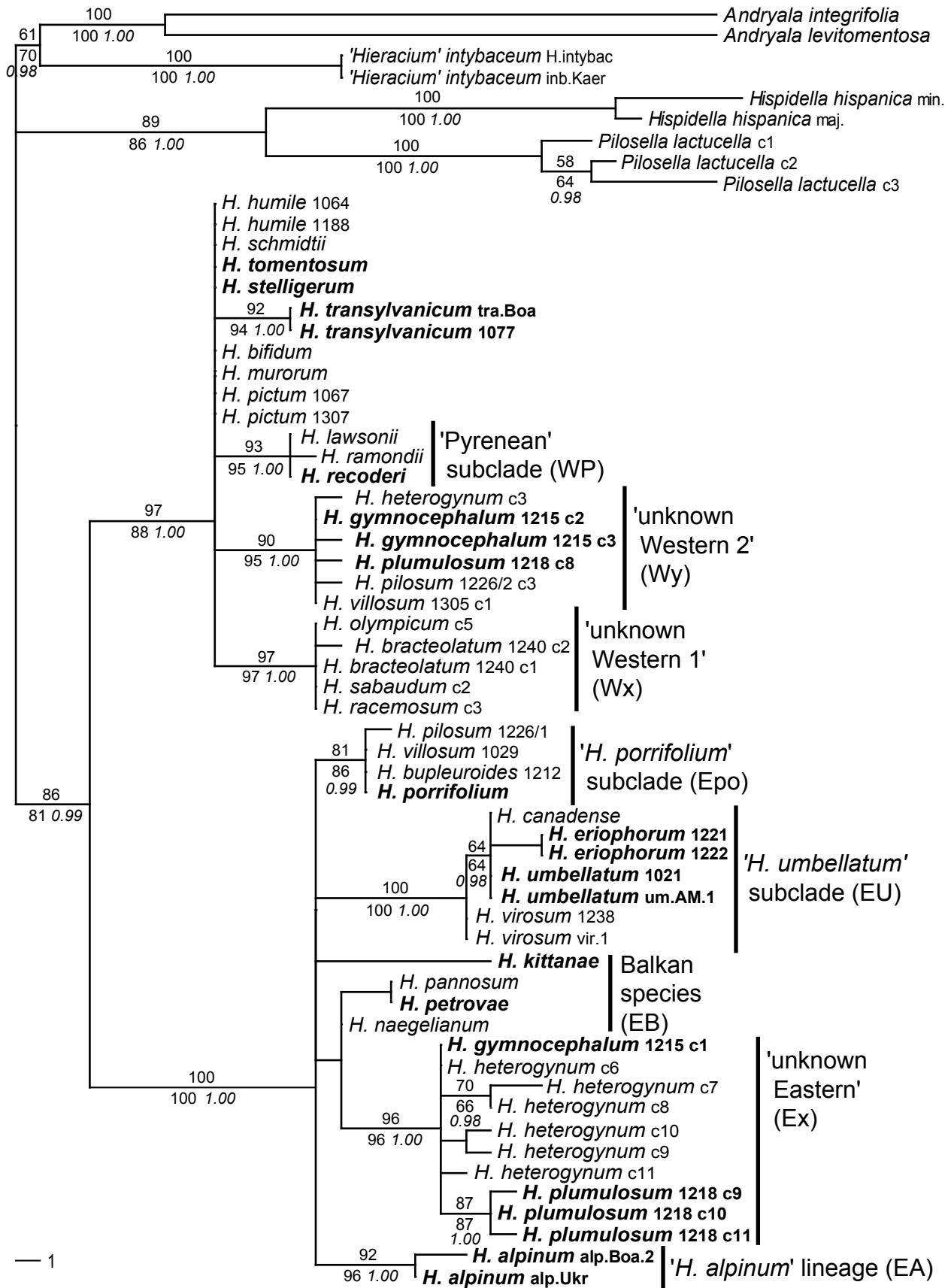

Supplement: Additional file 6 — ETS phylogeny with ribotypes present only in hybrids. The file contains the phylogenetic analyses on which Figure 5 is based. One out of 1402 equally parsimonious trees is shown (1752 steps, ri = 0.952, ci = 0.982; 182 variable characters of which 95 were parsimony informative) with bootstrap support indicated above the branches. The strict consensus tree topology corresponds to the branches with support values. Bootstrap values for ML and posterior probabilities for Bayesian analyses are given below the branches. Diploid Hieracium species are indicated in boldface. All lineages comprise diploids (the 'unknown Western 1' ribotype also occurs in low amounts in diploid H. lucidum which was not cloned). The Wx, Wy, and Ex lineages are represented by all non-recombinant clones of these ribotypes. Identical clones of the same accession were included only once (see also Additional file 2: Patterns of ETS recombination). The four hybrid accessions still maintained in Figure 2 were excluded, and also H. lachenalii and H. sparsum because of inferred chloroplast capture. Figure 5 uses the basic structure of this tree on which all inferred reticulation events were mapped. [file 1471-2148-9-239-S6.PDF]
